# Increased photodynamic therapy sensitization in tumors using a nitric oxide-based nanoplatform with ATP-production blocking capability

**DOI:** 10.7150/thno.52997

**Published:** 2021-01-01

**Authors:** Qinyanqiu Xiang, Bin Qiao, Yuanli Luo, Jin Cao, Kui Fan, Xinghua Hu, Lan Hao, Yang Cao, Qunxia Zhang, Zhigang Wang

**Affiliations:** 1 Institute of Ultrasound Imaging, Second Affiliated Hospital of Chongqing Medical University, Chongqing, 400010, P. R. China.; 2 Department of Nephrology, Second Affiliated Hospital of Chongqing Medical University, Chongqing, 400010, P. R. China.; 3 Department of Neurosurgery, Second Affiliated Hospital of Chongqing Medical University, Chongqing, 400010, P. R. China.

**Keywords:** photodynamic therapy, hypoxia relief, mitochondrial respiration, adenosine triphosphate, nitric oxide

## Abstract

Photodynamic therapy (PDT) efficacy in cancer cells is affected by sub-physiological hypoxia caused by dysregulated and “chaotic” tumor microvasculature. However, current traditional O_2_-replenishing strategies are undergoing their own intrinsic deficiencies. In addition, resistance mechanisms activated during PDT also lead the present situation far from satisfactory.

**Methods:** We propose a nitric oxide (NO)-based theranostic nanoplatform by using biocompatible poly-lactic-co-glycolic acid nanoparticles (PLGA NPs) as carriers, in which the outer polymeric layer embeds chlorin e6 (Ce6) and incorporates L-Arginine (L-Arg). This nanoplatform (L-Arg@Ce6@P NPs) can reduce hyperactive O_2_ metabolism of tumor cells by NO-mediated mitochondrial respiration inhibition, which should raise endogenous O_2_ tension to counteract hypoxia. Furthermore, NO can also hinder oxidative phosphorylation (OXPHOS) which should cause intracellular adenosine triphosphate (ATP) depletion, inhibiting tumor cells proliferation and turning cells more sensitive to PDT.

**Results:** When the L-Arg@Ce6@P NPs accumulate in solid tumors by the enhanced permeability and retention (EPR) effect, locally released L-Arg is oxidized by the abundant H_2_O_2_ to produce NO.* In vitro* experiments suggest that NO can retard hypoactive O_2_ metabolism and save intracellular O_2_ for enhancing PDT efficacy under NIR light irradiation. Also, lower intracellular ATP hinders proliferation of DNA, improving PDT sensitization. PDT phototherapeutic efficacy increased by combining these two complementary strategies* in vitro/in vivo*.

**Conclusion:** We show that this NO-based nanoplatform can be potentially used to alleviate hypoxia and sensitize tumor cells to amplify the efficacy of phototherapy guided by photoacoustic (PA) imaging.

## Introduction

Photodynamic therapy (PDT) is a potential therapeutic anti-cancer strategy that induces cell apoptosis by efficiently converting molecular O_2_ into cytotoxic reactive oxygen species (ROS) 1-3. However, PDT efficacy is compromised by heterogeneous hypoxic conditions caused by aberrant micro vascularization 4, 5. Considerable efforts have been devoted to achieve O_2_-replenishing strategies which mainly include the use of H_2_O_2_ decomposition materials and O_2_-loaded nanoparticles (NPs). However, nanomaterials used for H_2_O_2_ decomposition such as MnO_2_ NPs 6, 7, platinum NPs 8, 9 or Fenton reagents 10, 11 are affected by low levels of H_2_O_2_ 12 or react with H_2_O_2_ too slowly 13. Furthermore, O_2_ vehicles such as red blood cells 14, 15, hemoglobin 16, 17 or perfluorocarbon vesicles 18, 19 are affected by poor O_2_ loading or rapid O_2_ leakage 20. During PDT, these strategies cannot prevent the hypoxic environment formation that results from the hyperactive O_2_ metabolism prevalent in tumor cells 21.

Mitochondrial respiration includes aerobic glycolysis (“Warburg effect”) and mitochondrial aerobic respiration (Mito-AR). O_2_ is primarily consumed via the Mito-AR associated oxidative phosphorylation (OXPHOS) and the electron transport chain (ETC) 22. Heterogeneous hypoxic conditions in a tumor are mainly due to hyperactive O_2_ metabolism of Mito-AR in proliferating tumor cells 23. Thus, suppressing O_2_ consumption can effectively improve O_2_ tumor partial pressure for counteracting tumor hypoxia 24. Tamoxifen (TAM) 25, metformin (MET) 26 and atovaquone (ATO) 27 have been recently proposed to reduce O_2_ consumption in tumor cells and enhanced PDT. However, efficacy of these strategies is affected by resistance mechanisms activated during PDT 28, 29. Thus, there is an urgent need to develop an innovative strategy to increase oxygen partial pressure in tumors that also increases sensitivity of cancer cells to phototherapy.

Mitochondrial function integrity ensures that tumor cells maintain energy metabolism homeostasis 30. Adenosine triphosphate (ATP) is one of the most important energy supply substances generated via Mito-AR related OXPHOS 23, 31 and has become an emerging target for tumor theranostics 32. That severe ATP depletion can directly induce necrosis or apoptosis through DNA damage has been shown in many anticancer agents (e.g. cisplatin) 33. Moderate inhibition of ATP can effectively sensitize cells to chemotherapy 34, radiotherapy 35, PTT 36. More importantly, ATP is vital to support DNA replication and maintain cell proliferation 37. Blocking the respiratory chain can induce mitochondrial dysfunction and inhibit mitochondrial ATP synthesis. Decreased ATP levels can effectively induce cell cycle arrest 38-40 and increase sensitivity to PDT 37, 41, 42. Therefore, we propose herein a NO-based nanoplatform that could not only interrupt the synthesis of ATP for PDT sensitization, but also lead to O_2_ metabolism retardation. In the present study, we prepared this nanoplatform using biocompatible poly-lactic-co-glycolic acid nanoparticles (PLGA NPs) as carriers, where the outer polymeric layer of NPs embeds the photosensitizer chlorin e6 (Ce6) and incorporates L-Arginine (L-Arg). L-Arg is an effective hydrophilic NO donor that acts as a substrate for inducible nitric oxide synthases (iNOS) 43 or is oxidized by endogenous H_2_O_2_ 44 which in excess within the tumor region contributes to aberrant metabolism in cancer cells 45. Nitric oxide (NO) plays as a “star” gasotransmitter and induces a multitude of antitumor activities, which have been extensively applied to the synergism of PDT. For instance, Ji *et al.* 46 proved that NO could not only deplete intracellular glutathione (GSH), but also react with ROS to generate reactive nitrogen species (RNS) that enhance PDT. Dong* et al.* 47 reported NO could directly kill cancer cells at mM concentrations and improve PDT efficacy, although higher concentrations (> 1 mM) may cause NO poisoning 48, 49. Zhang *et al.* 50 proved that NO-induced by ROS could effectively sensitize PDT in hypoxia owing to the ability of NO to freely diffuse into deep hypoxic tumor sites.

**Scheme 1:**
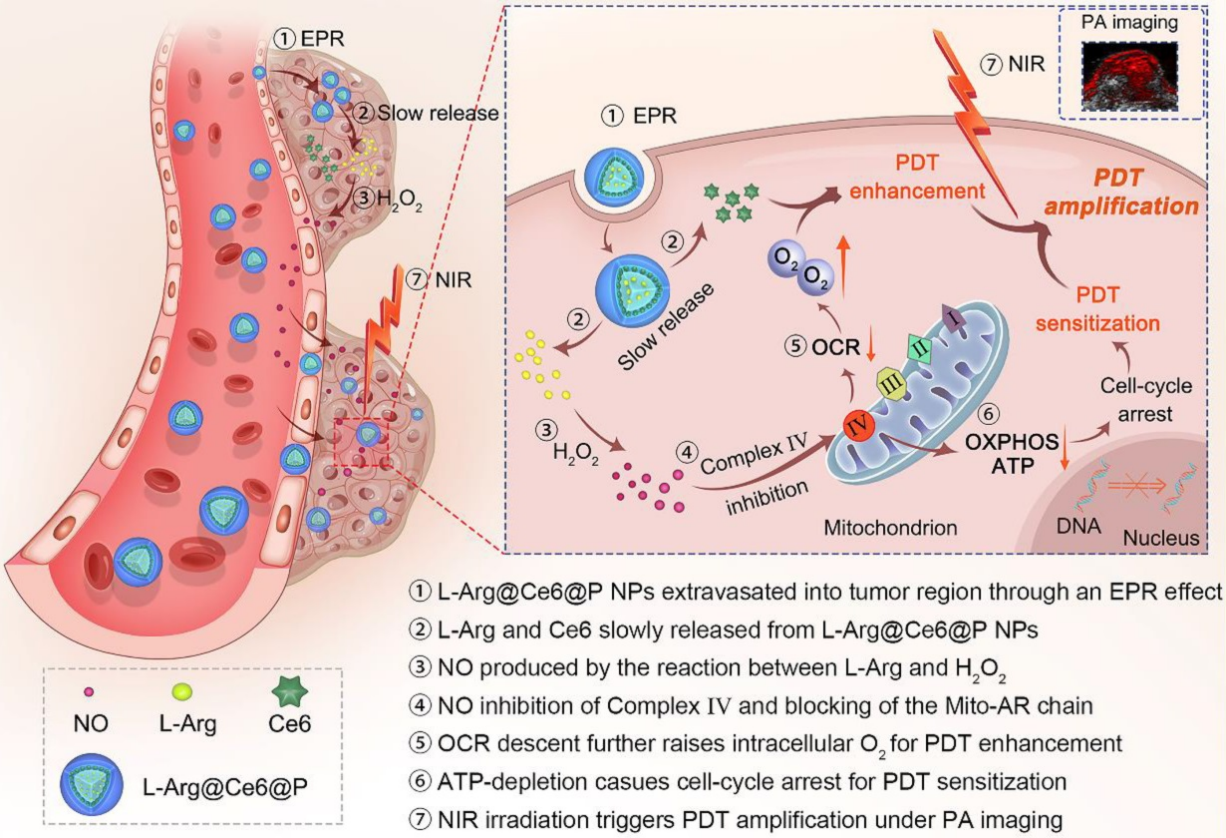
Schematic illustration of the L-Arg@Ce6@P NPs, the NO-based theranostic nanoplatform which amplifies PDT efficacy by hypoxia relief and ATP depletion.

In the present study, in contrast to the pathways shown above** (Scheme 1)**, when L-Arg@Ce6@P NPs accumulate in solid tumors by EPR effect 51, 52, locally released L-Arg is oxidized by the abundant H_2_O_2_ to produce NO. The NO level reached, nM to mM, does not kill the cell but inhibits the mitochondrial Complex Ⅳ (Cytochrome *c* oxidase, C*c*O) which accounts for 90% of O_2_ consumption and is essential for ATP production 53, 54. We propose that when NO diffuses freely within tumor tissue, Complex Ⅳ inhibition can retard hypoactive O_2_ metabolism and the increased O_2_ can enhance PDT efficacy. Also, lower intracellular ATP hinders proliferation of DNA, improving PDT sensitization. PDT phototherapeutic efficacy can be increased by combining these two complementary strategies. In addition, since the method uses Ce6, the nanoplatform acts as photoacoustic (PA) imaging contrast agent that can be used to monitor dynamic changes in the tumor, guiding the phototherapeutic process and leading to further optimization.

## Experimental Section

### Reagents

Biocompatible poly-lactic-co-glycolic acid (50:50, PLGA, MW: 12,000 Da) was obtained from Shandong Key Laboratory of Medical Polymer Materials (Shandong, China). Chlorin e6 (Ce6) was from J&K (Beijing, China) and L-Arginine from Aladdin-Reagent Ltd., Co. (Shanghai, China). Polyvinyl alcohol (PVA) was purchased from Sigma-Aldrich Chemical Co. (St Louis, USA). Other reagents kits were: chloroform (CHCl_3_) and isopropyl alcohol (Chongqing East Chemical Industry Ltd., Co., Chongqing, China), Singlet Oxygen Sensor Green (SOSG) (Invitrogen, Massachusetts, USA), hypoxia detection kit (Enzo Life Sciences Inc., Farmingdale, USA), Complex IV activity assay kit (Solarbio Science &Technology Ltd., Co., Beijing, China). Calcein-AM, propidium iodide (PI) and CCK-8 assays were from Dojindo Laboratories (Kumamoto, Japan). 3-Amino,4-aminomethyl-2',7'-difluorescein, diacetate (DAF-FM DA), Griess Reagent, JC-1 assay kit, ATP assay kit, 2,7-dichlorodihydrofluorescein diacetate (DCFH-DA), 4,6-diamidino-2-phenylindole (DAPI) were all obtained from Beyotime Biotechnology Ltd., Co. (Shanghai, China).

### Synthesis of L-Arg@Ce6@P NPs

The method of double emulsion (W/O/W) was used to synthesize L-Arg@Ce6@P NPs. Briefly, PLGA (50 mg) and Ce6 (2 mg) were dissolved in chloroform (2 mL), followed by addition of 400 µL of deionized water containing 40 mg L-Arg (100 mg/mL). An ultrasonic probe (Sonics & Materials, Inc., USA) was used to emulsify the mixture for primary emulsification. This primary emulsion was dispersed in 8 mL PVA solution (4% w/v) followed by a second sonication to form a W/O/W double-emulsion. The chloroform in the emulsion was removed by agitation with a magnetic stirrer for 2 h followed by centrifugation (10,000 rpm for 8 min) to obtain L-Arg@Ce6@P NPs. The integral L-Arg@Ce6@P NPs were finally stored at 4 °C for later use.

### Characterization of L-Arg@Ce6@P NPs

NP size distribution and Zeta-potential were measured using dynamic light scattering (DLS, Malvern Instruments, Malvern, UK). The mean particle size of L-Arg@Ce6@P NPs dissolved in either phosphate-buffered solution (PBS) or DMEM cell culture medium containing 10% serum was measured for different time periods (0, 3, 6, 12 and 24 h).The morphology and structure of L-Arg@Ce6@P NPs was monitored using transmission electron microscopy (TEM, Hitachi H-7600, Japan) and scanning electron microscopy (SEM, JEOL JEM-7800F). Optical absorption was determined with a UV-vis spectrophotometer (US-2550, Shimadzu, Japan). Different concentrations of free Ce6 were used to obtain a calibration curve using UV-Vis spectrometry, which was used to measure unloaded Ce6 in the supernatant.

To quantify loaded L-Arg in NPs, L-Arg@Ce6@P NPs were demulsified by dimethyl sulphoxide (DMSO) and L-Arg was detected by high-performance liquid chromatography (HPLC, Shimadzu LC-20AD, Japan): HPLC column (4.6 mm×150 mm, 5 μm); mobile phase: phosphate buffer/C_2_H_3_N (81/19, v/v); detection wavelength: 210 nm; flowrate: 1.0 mL/min. Loading content (LC) of L-Arg and Ce6, and Encapsulation efficiency (EE) of Ce6 were calculated according to 55, 56:

EE_Ce6_ = (weight of loaded Ce6 in NPs/total weight of Ce6) ×100%

LC_L-Arg/Ce6_ = (weight of loaded L-Arg or Ce6 in NPs/total weight of NPs) ×100%

### Cell culture

Human breast cancer line MDA-MB-231 cells were cultured in high glucose medium (DMEM, Gibico) containing 10% fetal bovine serum (FBS) and 1% antibiotics (100 U/mL penicillin and 100 μg/mL streptomycin) under a humidified atmosphere with 5% CO_2_ at 37 °C. To mimic the hypoxic environment, a simple and practicable physical treatment called liquid paraffin covering method was applied. The culture medium was replaced by serum-free DMEM when the system was covered.

### Cell uptake of L-Arg@Ce6@P NPs

The cellular uptake of L-Arg@Ce6@P NPs was investigated by CLSM (Nikon A1+R, Japan) and flow cytometry (BD FACSvantage SE, USA). Briefly, cells were seeded in 6-well plates at a density of 2×10^5^ cells per well and incubated for 24 h. Cells were further cultured in fresh DMEM medium containing L-Arg@Ce6@P NPs (Ce6:10 μg/mL) and incubated for 0.5, 1.5, 3 or 6 h. All cells were fixed in 4% formaldehyde for 20 min and washed with PBS. Cells were stained with DAPI (λex/ λem = 364 nm/454 nm) for 15 min. CLSM was performed to record the fluorescent images, and intracellular uptake of L-Arg@Ce6@P NPs (Ce6: λex/ λem = 488 nm/610 nm) at different time points was quantified by flow cytometry.

### Quantification of NO release by Griess Reagent

Griess reagent was used to detect released NO *in vitro*. 1 mL solution of L-Arg@Ce6@P NPs (20 mg/mL) or Ce6@P NPs (20 mg/mL) dissolved in PBS were dialyzed (cut-off MW: 2,500 Da) against 10 mL PBS (pH = 7.4) containing H_2_O_2_ (10 mM) and incubated at 37 °C. The standard curve of NO was obtained from NaNO_2_ in the kit. The NO released from NPs at different time points was quantified by a multimode reader (260-Bio, Thermo Fisher Scientific, USA) at 540 nm.

### Quantitation of NO release by DAF-FM DA

Released NO in cells was also observed using the fluorescent probe DAF-FM DA (λex/ λem = 495 nm/515 nm). Typically, MDA-MB-231 cells were seeded in 6-well plates at a density of 2×10^5^ cells per well and incubated for 24 h. After that, the medium was replaced by fresh DMEM medium containing PBS, L-Arg@Ce6@P NPs (Ce6:10 μg/mL) or Ce6@P NPs (Ce6:10 μg/mL) and cells were incubated for another 12 h. After this incubation cells were stained with DAF-FM DA for 20 min. After washing the residual DAF-FM DA, cells were collected and resuspended in PBS for quantitative determination via flow cytometry. Cells in plates were fixed by 4% formaldehyde followed by staining with DAPI for 15 min. Images were obtained by fluorescence microscopy (Olympus BX51, Japan).

### Determination of Cytochrome* c* Oxidase activity

Cytochrome *c* oxidase (C*c*O) activity was monitored using a Complex IV activity assay kit. Briefly, MDA-MB-231 cells were cultured for 24 h in cell culture bottles (2×10^6^ cells per bottle) with 8 mL medium per bottle. Then cells were exposed for 12 h to PBS, L-Arg@Ce6@P NPs (Ce6:10 μg/mL), Ce6@P NPs (Ce6:10 μg/mL) and free L-Arg (30 μg/mL). Following the manufacturer's protocol, the C*c*O activity was calculated.

### Evaluation of O_2_ metabolism level induced by NO

MDA-MB-231 cells were grown in 6-well plates and incubated for 24 h. After that, they were treated with PBS, L-Arg@Ce6@P NPs (Ce6:10 μg/mL) and Ce6@P NPs (Ce6:10 μg/mL) for 12 h in a normoxic environment. The culture medium was covered with 2 mL liquid paraffin and the dissolved oxygen (DO) in the medium was detected every 6 minutes using the dissolved oxygen meter (B949712069, Mettler-Toledo Instruments Co., Ltd.). Samples of medium alone and cells without covering were used as control.

### Detection of intracellular hypoxia level

Intracellular hypoxia levels were detected with a hypoxia detection kit. Typically, cells were grown in cell-culture dishes at a density of 1×10^5^ cells/dish for 24 h. Then cells were cultured in fresh DMEM medium containing either PBS, L-Arg@Ce6@P NPs (Ce6:10 μg/mL) or Ce6@P NPs (Ce6:10 μg/mL) for another 12 h. After this, they were covered or not with liquid paraffin for 2 h. Cells treated with DFO (a chemical hypoxia inducer) for 2 h were used as a positive control. All dishes were stained with hypoxia red detection reagent l (λex/ λem = 488 nm/590 nm) for 30 min. After washing with PBS, the dishes were observed by CLSM. Hypoxia levels were quantified by flow cytometry.

### Detection of mitochondrial depolarization

Changes of mitochondrial membrane potential (MMP, ΔΨm) were monitored using the mitochondrial dye JC-1. Firstly, MDA-MB-231 cells were grown in cell-culture dishes at a density of 1×10^5^ cells per dish and incubated for 24 h, followed by 12 h incubation with either PBS, L-Arg@Ce6@P NPs (Ce6:10 μg/mL) or Ce6@P NPs (Ce6:10 μg/mL). Cells treated with CCCP (MMP inhibitor, 10 µM) for 20 min were used as a positive control group. All cells were then cultured in fresh medium containing 1 mL JC-1 staining solution (10 μM) and incubated for 20 min in the dark. JC-1 aggregates (λex/ λem = 585 nm/590 nm) and JC-1 monomers (λex/λem = 514 nm/529 nm) were observed by CLMS. To quantify the MMP, cells treated as above were collected and suspended in 200 µL PBS for flow cytometry. Ψm was calculated as the JC-1 aggregate/monomer ratio.

### Determination of intracellular ATP level

Cellular ATP levels were measured with an ATP assay kit. First, we investigated whether free L-Arg could decrease cellular ATP levels. MDA-MB-231 cells were grown in 6-well plates with a density of 2×10^5^ per well and after 24 h incubation they were treated for 12 h with different concentrations of free L-Arg (5, 10, 20, 40 and 80 μg/mL). Finally, cells were lysed followed by centrifugation (12,000 g for 5 min) at 4 °C for luminometer detection. To measure the ATP level of cells treated with NPs, cells were incubated for 12 h with PBS, L-Arg@Ce6P@P NPs (Ce6:10 μg/mL) or Ce6@P NPs (Ce6:10 μg/mL) in a normoxic environment. The hypoxia group was covered with liquid paraffin for another 2 h. Finally, cells were collected and centrifuged, and the supernatant was used for RLU detection by a luminometer with multimode reader (260-Bio, Thermo Fisher Scientific, USA).

### Determination of cell cycle

The effect of NPs on the cell cycle was measured with flow cytometry. MDA-MB-231 cells were grown in 6-well plates at a density of 2×10^5^ per well. After 24 h incubation, cells were incubated for 24 h in either PBS, L-Arg@Ce6@P NPs (Ce6:10 μg/mL), Ce6@P NPs (Ce6:10 μg/mL) or free L-Arg (40 μg/mL) in a normoxic environment. After that, cells were collected and resuspended in 100 μL PBS followed with 900 μL of 75% ethanol, and finally detected via flow cytometry analysis.

### Determination of ROS levels

ROS levels *in vitro* were measured with SOSG (λex/λem = 504 nm/525 nm). Briefly, different concentrations of L-Arg@Ce6@P NPs and SOSG (5 μM) were added into cuvettes, followed by irradiation with a 660 nm NIR laser (Stone Laser, China) at a power density of 5 mW/cm^2^ for different time intervals. Fluorescence intensity changes of SOSG were observed by a multimode reader (260-Bio, Thermo Fisher Scientific, USA).

Intracellular ROS levels in MDA-MB-231 cells were detected with a typical fluorescent probe DCFH-A (λex/λem = 488 nm/530 nm). Cells were grown in cell-culture dishes with a density of 1×10^5^ per dish. After 24 h incubation, the medium was replaced by fresh DMEM medium containing either PBS, L-Arg@Ce6@P NPs (Ce6:10 μg/mL) or Ce6@P NPs (Ce6:10 μg/mL) and incubated for 12 h, with or without covering with liquid paraffin. After incubation, cells in the laser group received irradiation with a 660 nm laser at a power density of 5 mW/cm^2^ for 3 min. Samples were fixed in 4% formaldehyde for 20 min. Formaldehyde was removed and cells were dyed with DAPI for another 15 min. CLSM was used to observe intracellular ROS levels. Collected cells were used for flow cytometry analysis.

### *In vitro* anti-tumor efficacy

To estimate cytotoxicity *in vitro*, cell viabilities were determined by the CCK-8 method. First, the safety performance of free medicines and NPs were estimated. Typically, MDA-MB-231 cells were grown in a 96-well plate at a density of 1×10^4^ cells per well for 24 h. Then, different concentrations of L-Arg@Ce6@P NPs, Ce6@P NPs, free L-Arg and free Ce6 were added followed by 24 h incubation in the dark. PDT performance of NPs was estimated; cells were cultured in fresh DMEM medium containing different concentrations of L-Arg@Ce6@P NPs or Ce6@P NPs for 12 h, followed by a 2 h incubation in the dark whether with or without covering. Next, cells received a 660 nm laser irradiation at a power density of 5 mW/cm^2^ for 4 or 8 min, and cell viabilities were obtained by the CCK-8 method.

Finally, anti-tumor efficacy *in vitro* was estimated by live-dead cell staining. Typically, MDA-MB-231 cells were grown in 6-well plates or cell-culture dishes at a density of 2×10^5^ and 1×10^5^ per well respectively. After 24 h incubation, cells were incubated for 12 h with L-Arg@Ce6@P NPs (Ce6:10 μg/mL) or Ce6@P NPs (Ce6:10 μg/mL), and another 2 h in the dark, with or without covering. Next, the laser group cells received 660 nm laser irradiation at a power density of 5 mW/cm^2^ for 4 min. A dye solution of Calcein-AM (2 μM)/PI (4 μΜ) was used to quantify living/dead cells by CLSM. Treated cells were collected and resuspended in 200 μL PBS for quantitative detection via flow cytometry.

### Animals and tumor models

All BALB/c-nude mice were obtained from the Experimental Animal Center of Chongqing Medical University. All experiments and procedures performed on mice were based on the guidelines of the Institutional Animal Care and Use Committee. MDA-MB-231 tumor-bearing mice were established by subcutaneous administration of a cell suspension (1×10^6^ cells in 100 µL PBS for each mouse). The tumor size was measured using a vernier caliper, and tumor volumes were calculated as 0.5×length×width^2^.

### Biodistribution and PA imaging of L-Arg@Ce6@P NPs

Biodistribution of NPs *in vivo* was monitored by FL imaging. (λex/λem = 650 nm/700 nm) when tumor-bearing mice were i.v. injected with L-Arg@Ce6@P NPs (4 mg/mL, 200 µL). IndiGo 2.0.5.0 (Berthold Technologies, Germany) was used to obtain images at different time points (pre-, 6, 12 and 24 h post-injection) whereas fluorescence intensity was also analyzed by IndiGo software.

PA performance of L-Arg@Ce6@P NPs *in vitro/vivo* was evaluated with a Vevo LAZR Photoacoustic Imaging System (Visual Sonics Inc., Toronto, Canada). First, the maximum absorbance of L-Arg@Ce6@P NPs (1 mg/mL) was detected from 680 to 970 nm using 680 nm as excitation wavelength. Then, a calibration curve of PA values was obtained using several concentrations of L-Arg@Ce6@P NPs (1, 2, 4, 6 and 8 mg/mL) in saline solution. PA performance of L-Arg@Ce6@P NPs *in vivo* was further evaluated by PA imaging. Tumor-bearing mice were i.v. injected with L-Arg@Ce6@P NPs saline solution (4 mg/mL, 200 µL) and PA images were acquired at 0, 2, 4, 6, 12, 24 and 48 h after injection. PA imaging in oxy-hem mode was performed to assess tumor oxygenated hemoglobin levels after post-injection with L-Arg@Ce6@P NPs (4 mg/mL, 200 µL). Tumor inner oxyhemoglobin saturation (sO_2_ Avr Total) at different time points (0, 6, 12 and 24 h) was determined as the ratio of oxygenated (λ=850 nm) to deoxygenated (λ=750 nm) hemoglobin.

### Evaluation of tumor hypoxia/angiogenesis *in vivo*

Tumor-bearing mice were i.v. injected with saline solution containing Ce6@P NPs (4 mg/mL, 200 µL) or L-Arg@Ce6@P NPs (4 mg/mL, 200 µL) when tumor volume reached about 100 mm^3^. A group injected with saline solution (200 µL) was used as a control. Mice were sacrificed 24 h post-injection and tumors were harvested and cryo-sectioned onto slides. Fluorescence levels of HIF-1α (hypoxia level) and anti-CD_31_ (angiogenesis level) were observed and mean fluorescence intensity was analyzed with ImageJ software.

### Anti-tumor therapy efficacy *in vivo*

Tumor-bearing mice were distributed into five groups (six mice each): 1, Control; 2, Ce6@P NPs; 3, L-Arg@Ce6@P NPs; 4, Ce6@P NPs + Laser; 5, L-Arg@Ce6@P NPs + Laser. Saline solution containing L-Arg@Ce6@P NPs (4 mg/mL, 200 µL) or Ce6@P NPs (4 mg/mL, 200 µL) were i.v. injected to mice at day 0 and day 4, whereas mice i.v. injected with saline solution were used as the control group. Mice in groups 4 and 5 received laser irradiation (660 nm, 10 min, 95 mW/cm^2^) 24 h after injection, on the first and fifth day. In order to avoid photothermal effect and to ensure only effects due to PDT treatment, the temperature of the tumor region was monitored during irradiation with a Xenogen IVIS Spectrum imaging system (PerkinElmer, USA) so that temperature was always below 42 °C.

Mice body weights and tumor diameter were recorded in alternate days for the entire 18 days of observation. At day 6, one mouse in each group was sacrificed to collect tumors and major organs, which were examined for histological changes and apoptotic levels using Hematoxylin & Eosin (HE). These were complemented with immunofluorescence staining with Proliferating Cell Nuclear Antigen (PCNA) and terminal deoxynucleotidyl transferase-mediated dUTP-biotin nick end labeling (TUNEL) and mean fluorescence intensity was analyzed with ImageJ software.

### Biosafety of L-Arg@Ce6@P NPs

To investigate the toxicity of L-Arg@Ce6@P NPs *in vivo*, NPs (4 mg/mL, 200 µL) or saline solution were i.v. injected into BALB/c mice randomly divided into five groups (1, 3, 5, 7 and 14 d post-injection). At these days, major organs (heart, liver, spleen, lung and kidney) and blood samples were collected. Blood samples were used for different index analysis, including routine blood test, alanine aminotransferase (ALT), aspartate aminotransferase (AST), blood urea nitrogen (BUN), L-lactate dehydrogenase (LDH) and Creatinine (CREA). Staining of major organs with H&E was analyzed histologically.

### Statistical analysis

All data is expressed as mean ± SD and was analyzed with SPSS 22.0 software. Single Students' *t* and one-way ANOVA tests were used to detect statistical significance between pairs of groups or three or more groups, respectively. Significance levels are shown as *p < 0.05, **p < 0.01, ***p < 0.001.

## Results and Discussion

### Synthesis and characterization of L-Arg@Ce6@P

**Figure 1:**
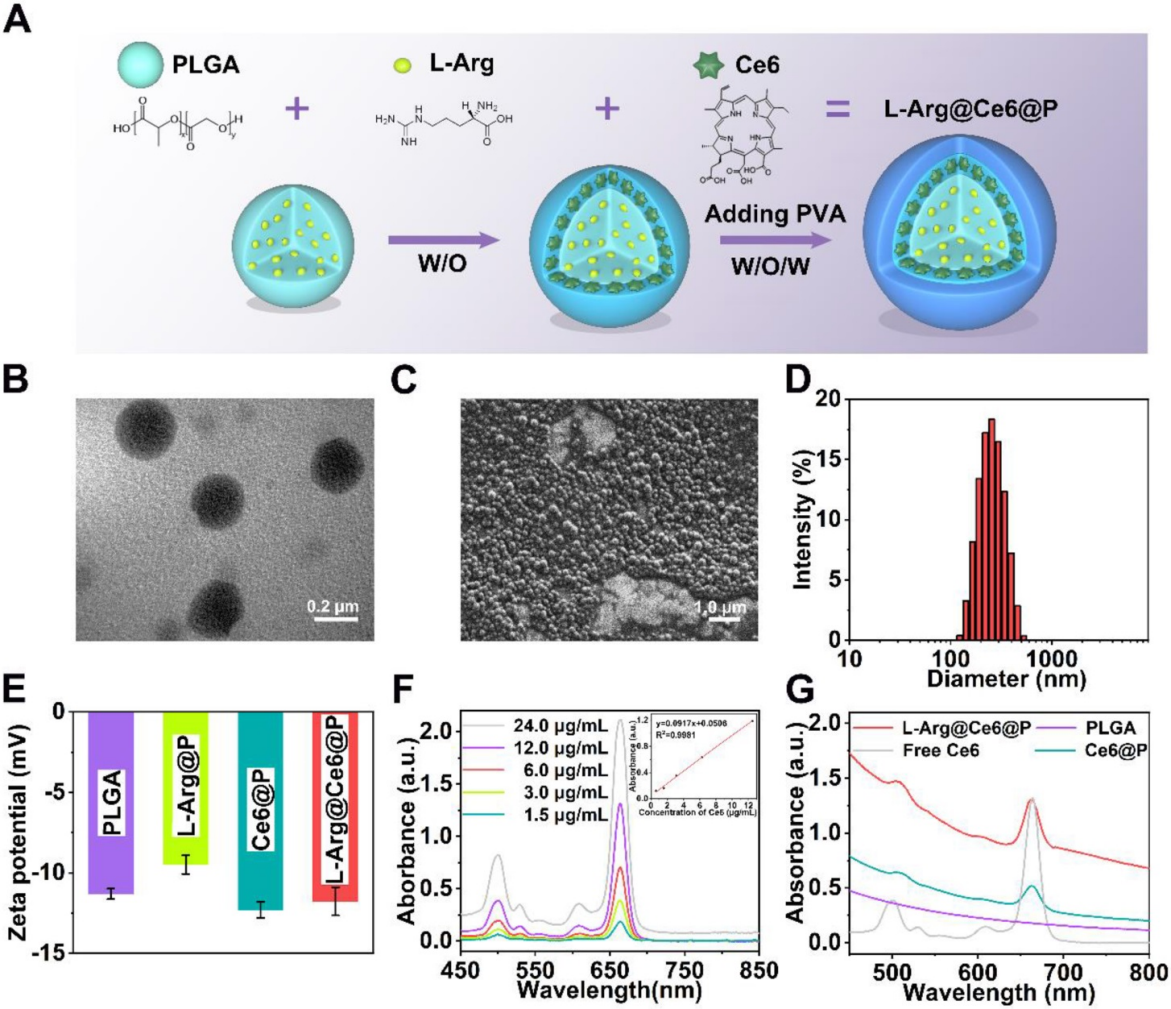
Morphology and characterization. (A) Schematic illustration of the synthesis process of L-Arg@Ce6@P NPs. (B) TEM image of L-Arg@Ce6@P NPs (scale bar: 0.2 µm). (C) SEM image of L-Arg@Ce6@P NPs (scale bar: 1.0 µm). (D) Size distribution of L-Arg@Ce6@P NPs as measured by DLS. (E) Zeta potential of different NPs (PLGA, L-Arg@P, Ce6@P, L-Arg@ Ce6@P). (n = 3). (F) UV-vis-NIR absorbance spectra of free Ce6 at elevated concentrations and the standard curve of free Ce6. (G) Absorbance spectra of different NPs (PLGA, Free Ce6, Ce6@P, L-Arg@Ce6@P) as recorded by UV-vis-NIR spectrophotometer.

L-Arg@Ce6@P NPs were synthetized using a double-emulsion approach **(Figure 1A)** 57-59. The obtained L-Arg@Ce6@P NPs were highly disperse as shown by transmission electron microscope (TEM)** (Figure 1B, S1A)**. L-Arg@Ce6@P NPs showed spherical morphology by scanning electron microscopy (SEM)** (Figure 1C)**. The average size of L-Arg@Ce6@P NPs obtained by DLS was around 242.2 nm **(Figure 1D)**, in agreement with the size obtained by TEM. DLS also showed that the size of PLGA, L-Arg@P and Ce6@P NPs also was narrowly distributed** (Figure S1B-D)** and centered at 200.3, 233.6 and 240.9 nm, respectively. The mean particle size of L-Arg@Ce6@P NPs did not show appreciable change within 24 h in either phosphate buffered saline (PBS) or DMEM cell culture medium containing 10% serum **(Figure S2A-B)**. The mean dispersion coefficient (PDI) was lower than 0.1 in both samples, indicating that the samples have excellent stability and can be applied to biological systems** (Figure S2C)**. The Zeta potential of PLGA NPs was -11.30 ± 0.40 mV whereas that of L-Arg@P NPs increased to -9.49 ± 0.71 mV, possibly due to the presence of positively charged arginine **(Figure 1E)**. However, the Zeta potential of the Ce6@P NPs decreased to -12.30 ± 0.61 mV owing to its shell being loaded with negatively charged Ce6. Finally, the Zeta potential of L-Arg@Ce6@P NPs was neutralized to -11.77 ± 1.07 mV.

The UV-vis-NIR spectrum of Ce6 in chloroform at different concentrations** (Figure 1F)** exhibited typical absorption peaks around 500 nm and 664 nm, and a calibration curve was obtained. Both Ce6@P NPs and L-Arg@Ce6@P NPs in PBS showed the characteristic absorption of free Ce6, indicating successful loading **(Figure 1G)**. Ce6 Encapsulation efficiency (EE) and Loading content (LC) were 61.2% and 2.2 wt %, respectively. A standard calibration curve of free L-Arg was obtained by HPLC **(Figure S3)**. The LC of L-Arg was 7.0 wt %.

### Cell uptake of L-Arg@Ce6@P NPs and NO detection

**Figure 2:**
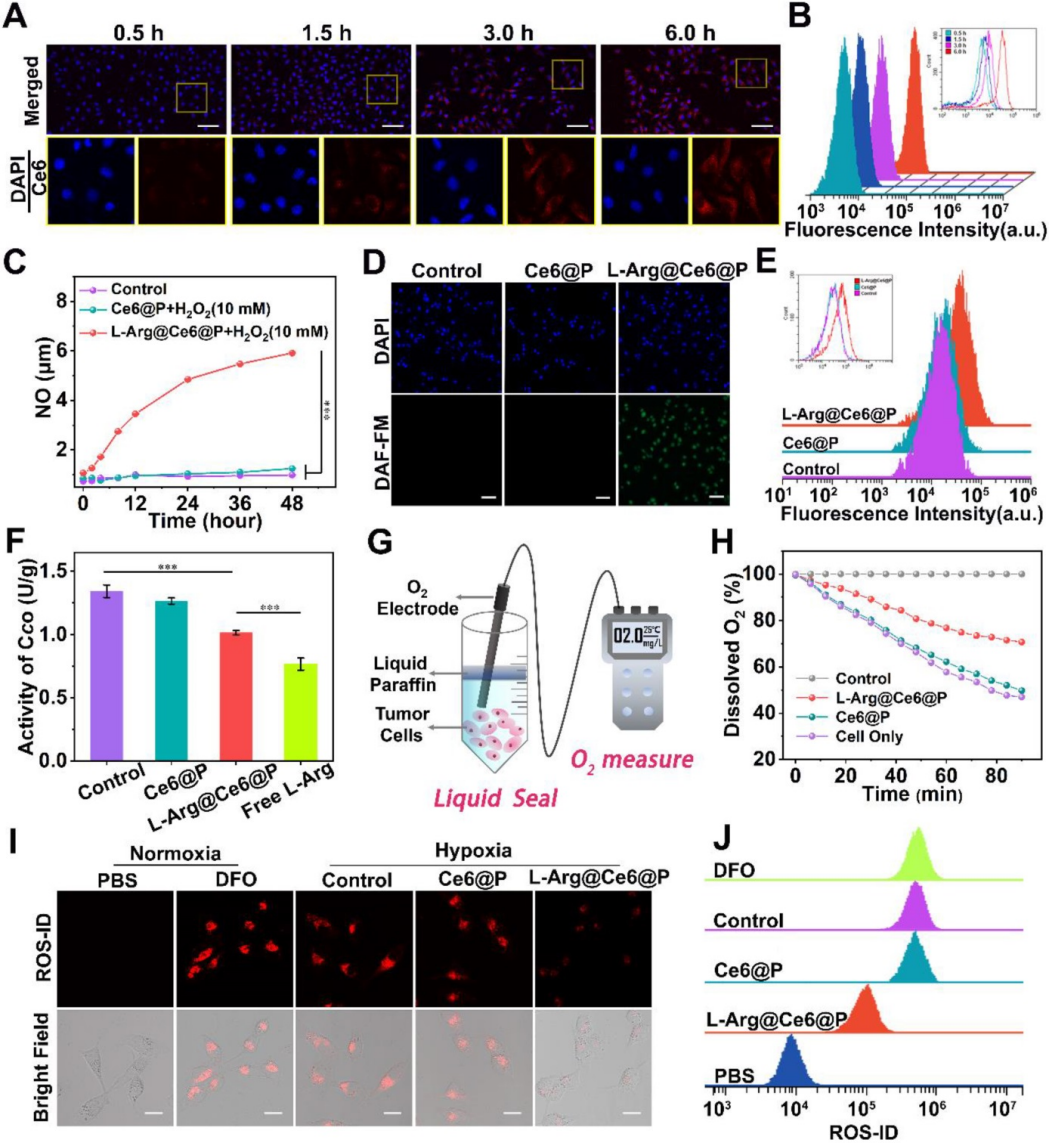
Cells uptake of NPs and NO-mediated hypoxia relief. (A-B) Intracellular uptake of L-Arg@Ce6@P NPs as observed by CLSM and quantified by flow-cytometry analysis. (Scale bar: 50 µm). (C) Cumulative NO release from NPs. (n = 3, ***p < 0.001). (D-E) Cells stained with DAF-FM DA observed by fluorescence microscope and quantified by flow-cytometry analysis. (Scale bar: 50 µm). (F) Activity of cytochrome *c* oxidase (C*c*O) after different treatments. (n = 3, ***p < 0.001). (G) Schematic illustration about measuring the O_2_ consumption of different groups. (H) Relative dissolved oxygen (DO) content changes in the cell medium of different groups. (I-J) Cells stained with ROS-ID in different groups observed by CLSM and quantified by flow-cytometry. (Scale bar: 20 µm).

To explore the efficiency of cellular uptake for L-Arg@Ce6@P NPs, the behavior of NPs was studied by Laser Confocal Scanning Microscopic (CLSM) and flow cytometric analysis. The red fluorescent signal from Ce6 was observed in MDA-MB-231 cells at 3 h post-coincubation** (Figure 2A)**. Quantitative analysis also illustrated the same phenomenon, indicating cellular uptake efficiency of more than 80% at 3 h **(Figure 2B)**. These results suggest efficient cell internalization of L-Arg@Ce6@P NPs, possibly because of their nanoscale dimensions and good biocompatibility.

The NO production of NPs involved two steps: (1) L-Arg release from L-Arg@Ce6@P NPs and (2) reaction of L-Arg with endogenously inducible nitric oxide synthases (iNOS) 43, or with H_2_O_2_ 60, 61, to produce NO. To verify whether L-Arg reacted with H_2_O_2_, the generated NO was measured using a Griess assay, with a standard curve obtained from the kit** (Figure S4).** With increasing water bath time, L-Arg@Ce6@P NPs react with H_2_O_2_ to produce significantly more NO than the other two groups **(Figure 2C)**, demonstrating that L-Arg@Ce6@P NPs can potentially interfere with mitochondrial O_2_ and energy metabolism. Then, intracellular NO levels were monitored with the fluorescent probe DAF-FM DA. Cell nuclei were stained with DAPI (blue fluorescence) and the green fluorescence of DAF-FM DA was visualized on samples exposed to L-Arg@Ce6@P NPs **(Figure 2D)**. Flow cytometry quantitative analysis also confirmed the generation of NO from L-Arg@Ce6@P NPs **(Figure 2E)**. The trend in fluorescence quantification** (Figure S5)** was consistent with the findings above.

### Mitochondria O_2_ metabolism retardation caused by NO

NO inhibits the activity of cytochrome *c* oxidase (C*c*O) in Complex IV by competitive binding to CcO O_2_-binding site 53, 54. The activity of CcO can be monitored with a Complex IV activity assay kit. A 12 h incubation with either free L-Arg or L-Arg@Ce6@P NPs decreased cellular CcO activity to 0.77 U/g and 1.02 U/g, respectively (1.02 is 75.65% of the control group) **(Figure 2F)**. This shows that both treatments effectively inhibit CcO activity on Mito-AR chain, a prerequisite to increase intracellular O_2_ for enhancing PDT.

Complex IV is responsible for almost 90% of O_2_ consumption 62, 63 therefore we hypothesized that NO can increase intracellular O_2_ after treatment with L-Arg@Ce6@P NPs. Consumption of O_2_ in a cell culture medium can be monitored by changes in dissolved oxygen (DO) detected with an oxygen electrode 26** (Figure 2G)**. Compared to the control group, DO of cells treated for 90 min with PBS or Ce6@P NPs experienced a sharp decrease to 46.9% and 49.7% respectively** (Figure 2H)**. In cells treated with L-Arg@Ce6@P NPs, the reduction in DO was slow and reached 70.8% of the control. Overall, these data show that L-Arg@Ce6@P NPs can effectively reduce mitochondria O_2_ consumption in cancer cells, counteracting hypoxia. To verify this, intracellular hypoxia status was monitored with a hypoxia detection kit. Cells treated with Ce6@P NPs and control group under hypoxia showed a strong red fluorescence, similar to cells treated with DFO (the positive control group) **(Figure 2I)**, whereas fluorescence intensity was markedly lower in cells treated with L-Arg@Ce6@P NPs. These results are consistent with flow analysis **(Figure 2J)** and fluorescence quantification **(Figure S6)** obtained from Image J. Thus, L-Arg@Ce6@P NPs relieves hypoxia inside cells.

### Mitochondria energy metabolism interference caused by NO

**Figure 3:**
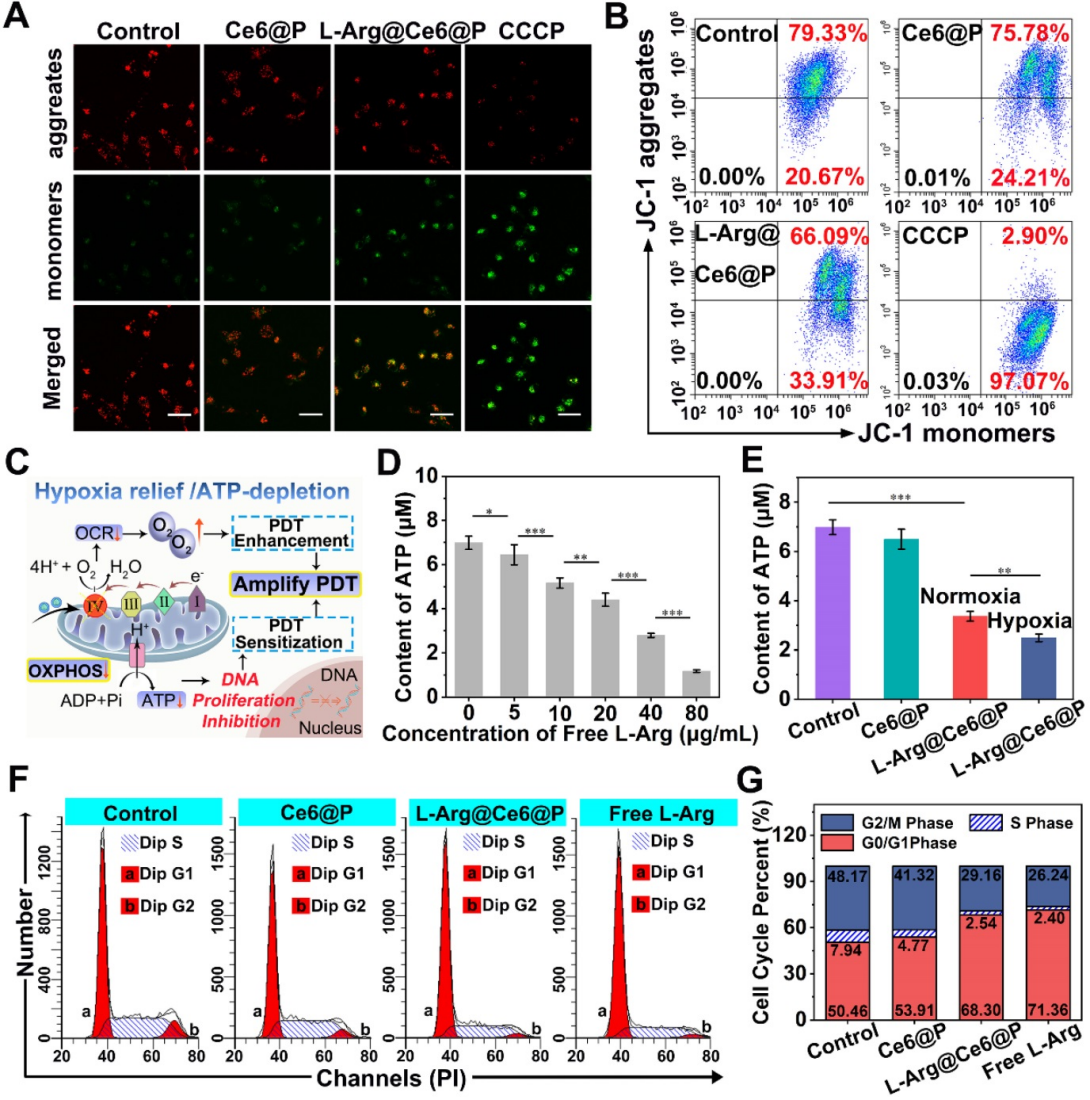
ATP-depletion and effects on cells. (A) Mitochondria membrane potential changes of cells after different treatments observed by CLSM. Cells treated with CCCP (ETC inhibitor) as the positive control group. (scale bar: 50 µm). (B) Flow-cytometry-based JC-1 assay as a measure of mitochondrial depolarization. (C) Schematic illustration of hypoxia relief and ATP-depletion for amplifying PDT. (D) Content of ATP after incubating with different concentrations of free L-Arg. (n = 3, *p < 0.05, **p < 0.01, ***p < 0.001). (E) Content of ATP after different treatments. (n = 3, **p < 0.01, ***p < 0.001). (F) Cell cycle of cells after different treatments. (G) The percentage of each phases on cell cycle in different groups.

Mitochondrial OXPHOS is the main contributor to cellular energy 30, where Complex IV acts as a proton pump. When NO inhibits Complex IV, electron transfer blockage causes mitochondrial dysfunction 62, 63, represented by changes of mitochondrial membrane potential (MMP, ΔΨm) and observed with the mitochondrial dye JC-1 64. With high Ψm, JC-1 aggregates deliver red fluorescence, whereas at low Ψm, JC-1 exists as monomers that produce green fluorescence. Whereas in cells treated with PBS or Ce6@P NPs JC-1 aggregates accumulated at the mitochondrial membrane and displayed brightly red fluorescence** (Figure 3A)**, the positive control group treated with CCCP displayed a strong green fluorescence due to JC-1 monomers. Cells treated with L-Arg@Ce6@P NPs experienced Ψm reduction and displayed enhanced green fluorescence and faint red fluorescence, indicating MMP depolarization. A quantification of JC-1 aggregates and JC-1 monomers was obtained from flow cytometry, and the extent of mitochondrial depolarization was calculated as the ratio of red to green fluorescence intensity. Although there were small changes in the Ce6@P group due to the weak toxicity of photosensitizers, Ψm in the L-Arg@Ce6@P group was reduced to 49.9% compared with the control group **(Figure 3B, S7)**. These results confirm that L-Arg@Ce6@P NPs reduce the integrity and function of mitochondria and can be used to amplify PDT.

It has been reported that NO can effectively downregulate ATP levels 65, mostly due to the pivotal role of CcO in ATP production 62, 63. Therefore, we hypothesized that L-Arg@Ce6@P NPs can block OXPHOS and inhibit ATP synthesis** (Figure 3C)**. To demonstrate that free L-Arg can block ATP production we used a calibration curve of ATP **(Figure S8)**. ATP levels decreased with increasing free L-Arg content, indicating that free L-Arg can effectively impede ATP production in cancer cells **(Figure 3D)**. ATP levels in the control and Ce6@P groups remained relatively high **(Figure 3E)**, but it was lower in both L-Arg@Ce6@P groups. Specifically, the ATP levels in the hypoxia group were significantly lower than in the normoxia group, about 3.4 μM to 2.5 μM, suggesting that NO inhibits ATP synthesis more at lower O_2_ concentrations 66.

Since mitochondrial ATP synthesis is essential for tumor cell growth, division, proliferation and metastasis 23, 37, we hypothesized that NO-induced ATP-depletion can inhibit cell proliferation reflected in the cell cycle. Compared with the control group, cells exposed to free L-Arg showed increased proportion of G0/G1 phase and lower proportion of S phase **(Figure 3F, 3G)**. Since the replication and synthesis of DNA takes place at the S phase, this suggests that NO can block the cell cycle in the G_0_/G_1_ phase, consistent with a previous study 67. We note that even if there was a small decrease of S phase in cells treated with Ce6@P, caused by the weak toxicity of the photosensitizers, in the L-Arg@Ce6@P group the proportion of G_0_/G_1_ phase was significantly higher, and both S and G_2_/M phases were much lower. These results suggest that L-Arg@Ce6@P NPs affects cell cycle and DNA replication due to decrease in ATP levels. These effects can aid in PDT sensitization.

### PDT performance *in vitro*

**Figure 4:**
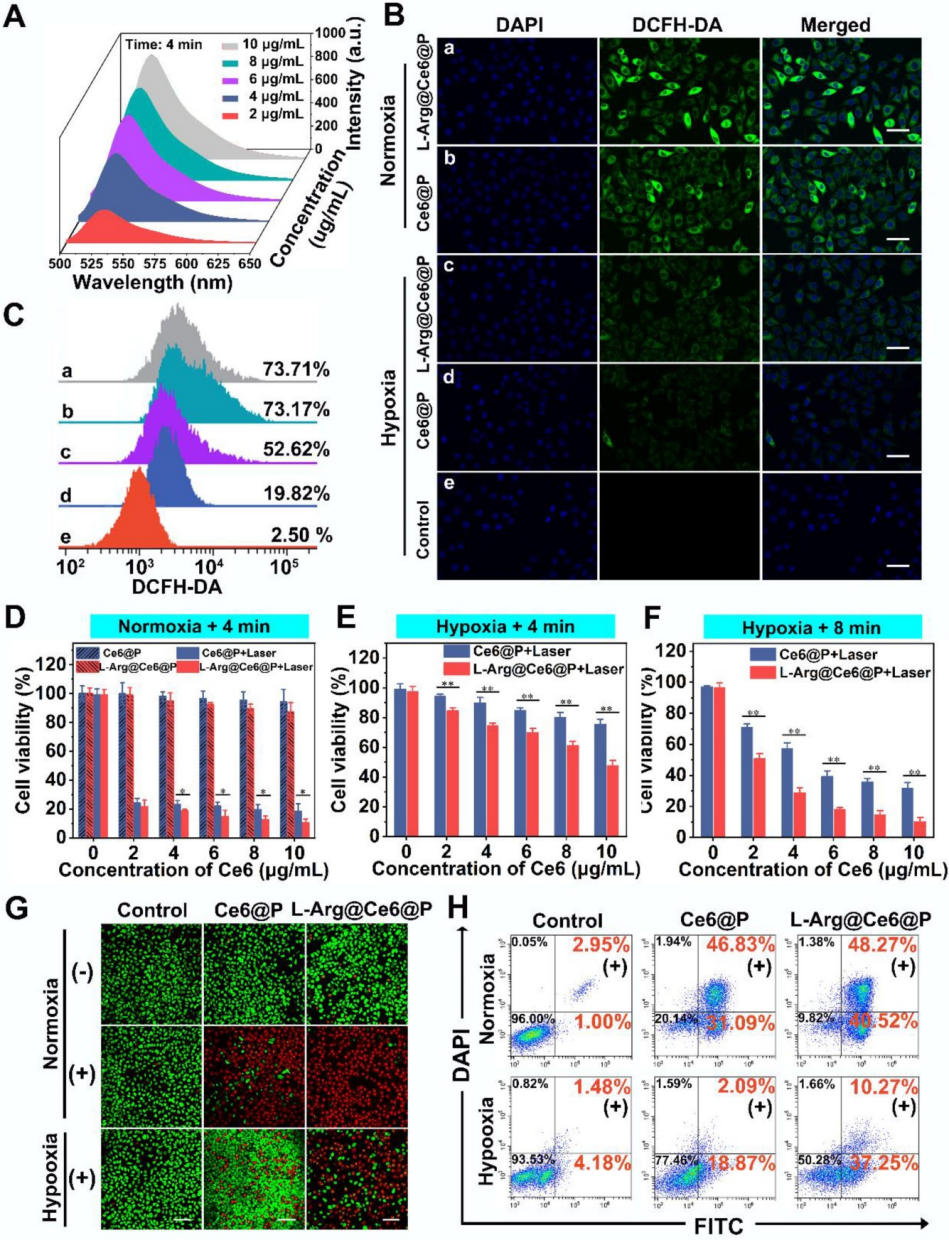
*In vitro* PDT of cancer cells. (A) Concentration-dependent ^1^O_2_ generation of L-Arg@Ce6@P NPs irradiated by 660 nm laser (5 mW cm^-2^) for 4 min. (B-C) Intracellular ROS level observed by CLSM and quantified by flow-cytometry analysis after different treatments (5 mW cm^-2^ ×3 min). (scale bar: 50 µm). (D-F) Relative cell viability of cells after different treatments (5 mW cm^-2^ ×4 min or 8 min). (n = 3, *p < 0.05, **p < 0.01). (G-H) Cells stained with Calcein-AM/PI staining after different treatments (5 mW cm^-2^ ×4 min) observed by CLSM and apoptosis quantified by flow-cytometry analysis. (**-**) means without laser irradiation, (**+**) means with laser irradiation. (Scale bar: 50 µm).

The fluorescence intensity of SOSG, an indicator to investigate ROS levels *in vitro*, increased in a concentration-dependent manner after mixing with L-Arg@Ce6@P NPs (Ce6:10 µg/mL) and receiving a laser irradiation (660 nm, 5 mW/cm^2^, 4 min or 8 min)** (Figure 4A, S9)**. Additionally, a time-dependent fluorescence increase was observed at the various concentrations of Ce6 (2, 4, 6, 8 and 10 µg/mL) under different irradiation duration** (Figure S10A-E)**.

The intracellular^ 1^O_2_ generation capability of L-Arg@Ce6@P NPs was detected by DCFH-DA under normoxic and hypoxic environments. Upon 660 nm laser irradiation (5 mW/cm^2^) for 3 min, cells treated with L-Arg@Ce6@P NPs or Ce6@P NPs showed similarly strong green fluorescence under normoxia whereas under oxygen-deficient conditions, L-Arg@Ce6@P group was brighter than the Ce6@P group** (Figure 4B)**. Quantitative results obtained from flow cytometry were consistent with the above findings observed by CLMS **(Figure 4C)**. Under normoxia, cells treated with L-Arg@Ce6@P NPs and Ce6@P NPs exhibited similar percentages of ROS production (73.71% and 73.17%, respectively). However, in hypoxia conditions, ROS production in cells treated with L-Arg@Ce6@P NPs was 2.7-fold higher than in cells treated with Ce6@P NPs (52.62% and 19.82%, respectively), whereas cells treated with PBS without laser exhibited negligible fluorescence (2.50%). These results demonstrate that L-Arg@Ce6@P NPs produce more ROS and lay a solid foundation for achieving PDT enhancement under hypoxia.

### Anti-tumor efficacy *in vitro*

We then evaluated the PDT therapeutic effect of NPs *in vitro*. Cell viability was high when treated with increasing free L-Arg and free Ce6 in PBS** (Figure S11)**. For the Ce6 group it was more than 80%, even when the concentration exceeded 25 µg/mL, far in excess of therapeutic concentrations (10 µg/mL). Under normoxia, cell viability of the two groups without laser irradiation showed no significant differences and persisted above 85% at therapeutic concentrations **(Figure 4D)**. By comparison, when the two groups were exposed to 4 min laser irradiation **(Figure 4D)** cell viability was dramatically reduced. The L-Arg@Ce6@P group exhibited more cell lethality than the Ce6@P group, indicating that even though the ROS production of the two groups at normoxia is similar, NO caused ATP-depletion in the L-Arg@Ce6@P group, effectively sensitizing cells to PDT and causing more cell apoptosis.

In hypoxia, after irradiation for 4 min, both L-Arg@Ce6@P and Ce6@P showed only a mild anti-cancer effect **(Figure 4E)**, possibly because insufficient O_2_ supply. Prolonging the irradiation time to 8 min under hypoxia **(Figure 4F)**, cell viability in the two groups significantly decreased, whereas cell lethality in the L-Arg@Ce6@P NPs group was significantly higher. The differences between the two groups in hypoxia conditions (whether after 4 min or 8 min laser irradiaton) were significantly higher than the one observed between two groups in normoxia. Under the low O_2_ levels, the L-Arg@Ce6@P group exhibited more intense phototoxicity than the Ce6@P group. This can be explained by the alleviation of hypoxia by L-Arg@Ce6@P NPs and by the PDT sensitization caused by ATP depletion, combination of these effects results in enhancement of PDT.

The findings above were further confirmed by CLSM observation of cells with live/dead cell staining **(Figure 4G)**. Under normoxia, the three groups without laser treatment showed strong green florescence. When cells were exposed to a laser for 4 min, the red florescence of apoptotic cells in L-Arg@Ce6@P or Ce6@P groups increased significantly, reaching apoptosis of 89% and 78%, respectively **(Figure 4H)**. This is attributed to PDT sensitization caused by ATP depletion. In contrast, when cells were cultured in hypoxia and exposed to a laser for 4 min, red florescence was attenuated, although that of L-Arg@Ce6@P group was higher than the Ce6@P group. The cell apoptosis results were consistent with this, with 48% and 21% in L-Arg@Ce6@P and Ce6@P groups, respectively. This difference (27%) is nearly 2.4 times the difference observed under normoxia (11%), suggesting that L-Arg@Ce6@P is far superior in killing tumor cells as a result of hypoxia relief and PDT sensitization induced by ATP depletion.

### Biodistribution and PA imaging of L-Arg@Ce6@P NPs

Fluorescence (FL) and Photoacoustic (PA) imaging were used to evaluate the behavior of NPs *in vivo*. First, FL imaging was performed to verify whether L-Arg@Ce6@P NPs could accumulate in tumor region by EPR. The FL signal of Ce6 within the tumor region reached its peak at 24 h post-injection **(Figure S12)**, clearly indicating that NPs effectively reached the tumor site.

**Figure 5:**
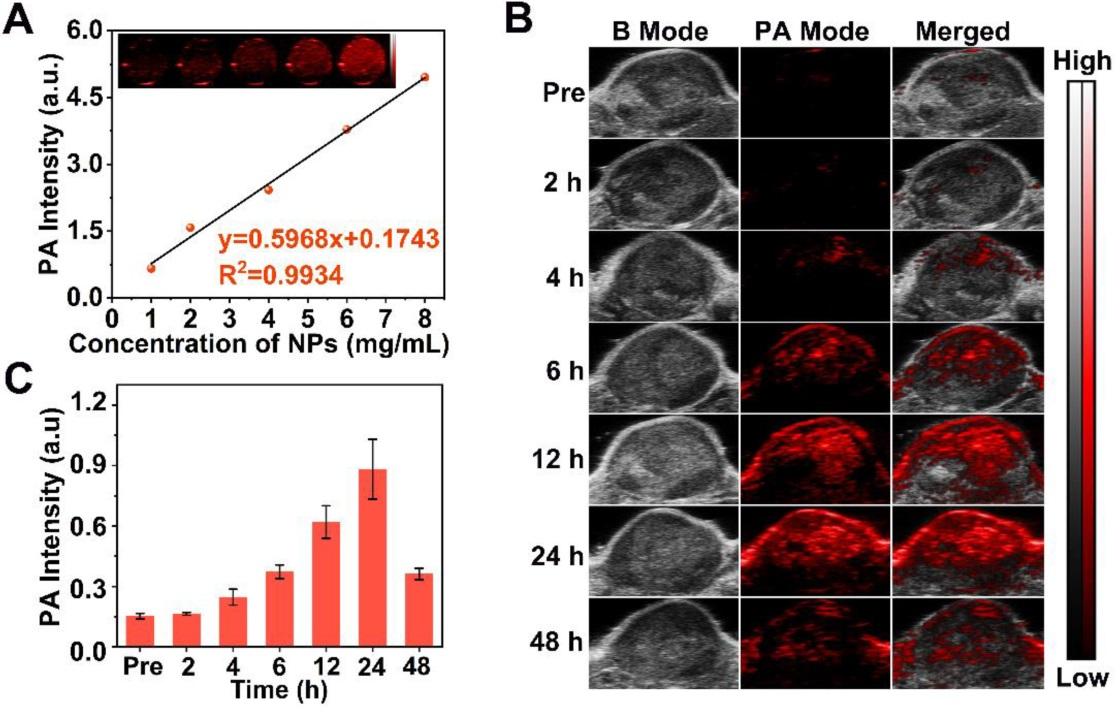
PA imaging of L-Arg@Ce6@P NPs *in vitro* and *in vivo*. (A) *In vitro* PA contrast images and PA values of L-Arg@Ce6@P NPs at different concentrations. (B) *In vivo* PA images of tumors in tumor-bearing mice after i.v. injection of L-Arg@Ce6@P NPs at different time points. (C) Changes of PA-signal intensities within tumor regions at corresponding time points. (n = 3).

Since PA imaging benefits from superb contrast, exceptional spatial resolution and excellent sensitivity in biological tissue 68, 69 and Ce6 is effective as PA contrast agent 46, we used this method to image L-Arg@Ce6@P NPs. The highest PA intensity of L-Arg@Ce6@P NPs was located at 680 nm **(Figure S13)**, which was used as excitation wavelength. The PA signal of NPs was clearly dose-dependent with an excellent linear relationship **(Figure 5A)**, increasing from 0.66 to 4.96 with NPs concentration (1, 2, 4, 6 and 8 mg/mL). Then we evaluated PA imaging performance of NPs *in vivo*. The PA signal within the tumor region was observed at 4 h post-injection **(Figure 5B)** and increased in a time-dependent manner, finally reaching a peak at 24 h post-injection. The quantitative analysis showed a similar trend **(Figure 5C)**. Overall, PA imaging of L-Arg@Ce6@P NPs *in vivo* suggest that NPs accumulate in tumor tissue and help in imaging, providing superb information on tumor microstructure.

### Hypoxia relief induced by NO *in vivo*

**Figure 6:**
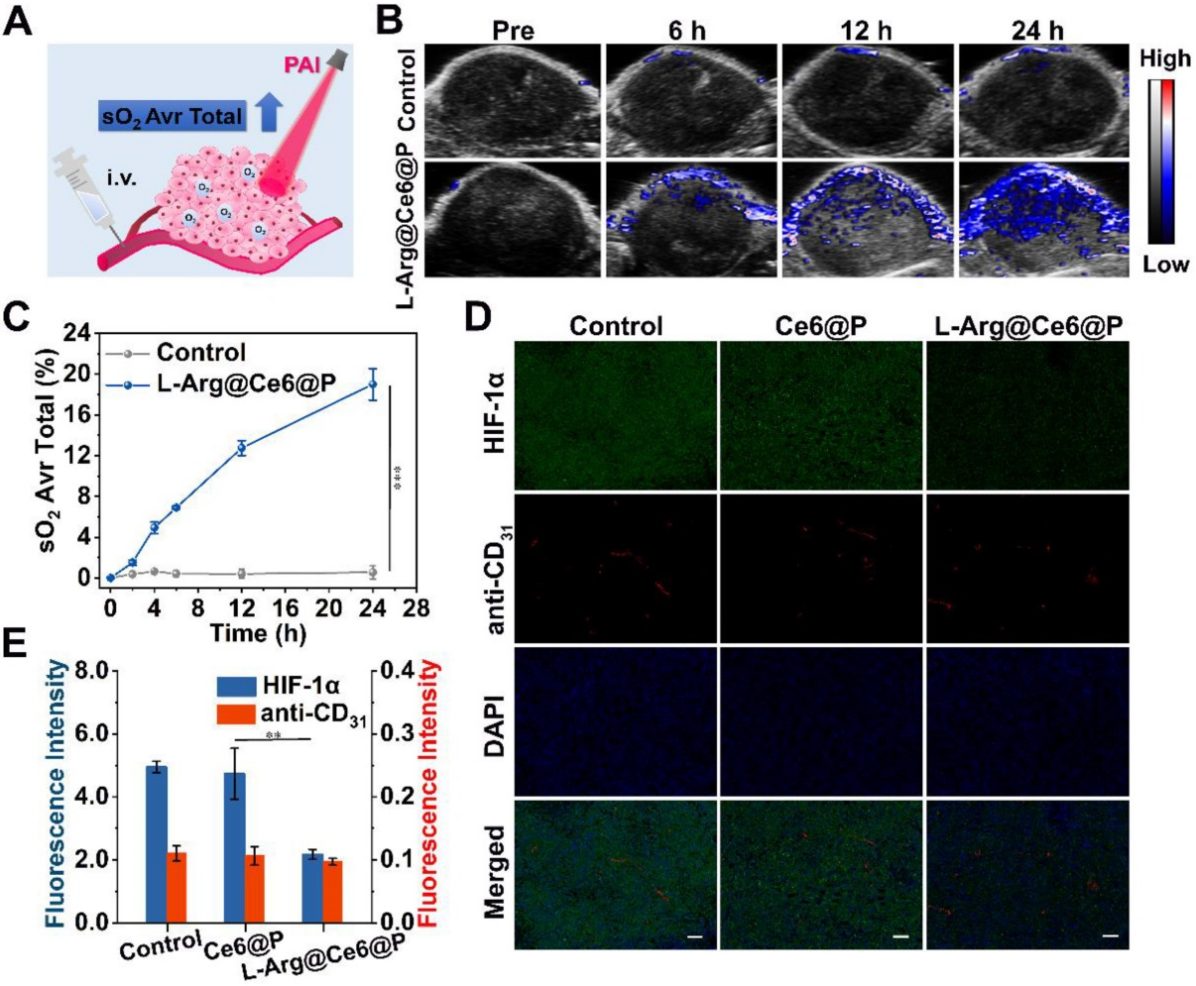
Tumor-hypoxia status *in vivo*. (A) Schematic illustration of measuring oxyhemoglobin saturation by PA imaging. (B) PA images of tumor sites in oxy-hemoglobin mode at different time points. (C) Quantification of oxyhemoglobin saturation at tumor sites by measuring the ratios of oxygenated hemoglobin (λ = 850 nm) and deoxygenated hemoglobin (λ = 750 nm). (n = 3, ***p < 0.001). (D) Immunofluorescent images of tumor slices stained by the hypoxia probe and blood vessel probe. (scale bar: 50 µm). (E) Mean Fluorescence intensity quantitative analysis of HIF-α and anti-CD_31_. (n = 3, **p < 0.01).

To further verify the assumption that L-Arg@Ce6@P NPs alleviate hypoxia *in vivo*, the oxyhemoglobin signal captured by the PA imaging system was used to monitor the dynamic alteration of hypoxic levels in the tumor** (Figure 6A)**. Oxyhemoglobin signal intensity within tumor tissues at 12 h post-injection with L-Arg@Ce6@P NPs was higher than in control group **(Figure 6B)**. The quantitative analysis indicated that oxyhemoglobin intensity in the L-Arg@Ce6@P group increased from 2.7% to 19.0% at 24 h post-injection, whereas almost no change was observed in the group treated with saline** (Figure 6C)**.

To further confirm the ability of L-Arg@Ce6@P NPs in relieving tumor hypoxia, we used HIF-1α immunofluorescence staining **(Figure 6D)**. Tumor slices treated with saline showed green fluorescence, suggesting intra-tumor hypoxia, whereas the tumor region after i.v. injection with L-Arg@Ce6@P NPs presented just faint green fluorescence. Semi-quantitative analysis of the hypoxia areas** (Figure 6E)** suggested that the fluorescence intensity in tumor tissue was reduced 56% after injection with L-Arg@Ce6@P NPs, compared with the control group. However, blood vessel density was similar for the two groups. Thus, these results validate the assumption that L-Arg@Ce6@P NPs relieves tumor inner hypoxia status, and lay the foundation for enhancing PDT.

### Anti-tumor efficacy* in vivo*

**Figure 7:**
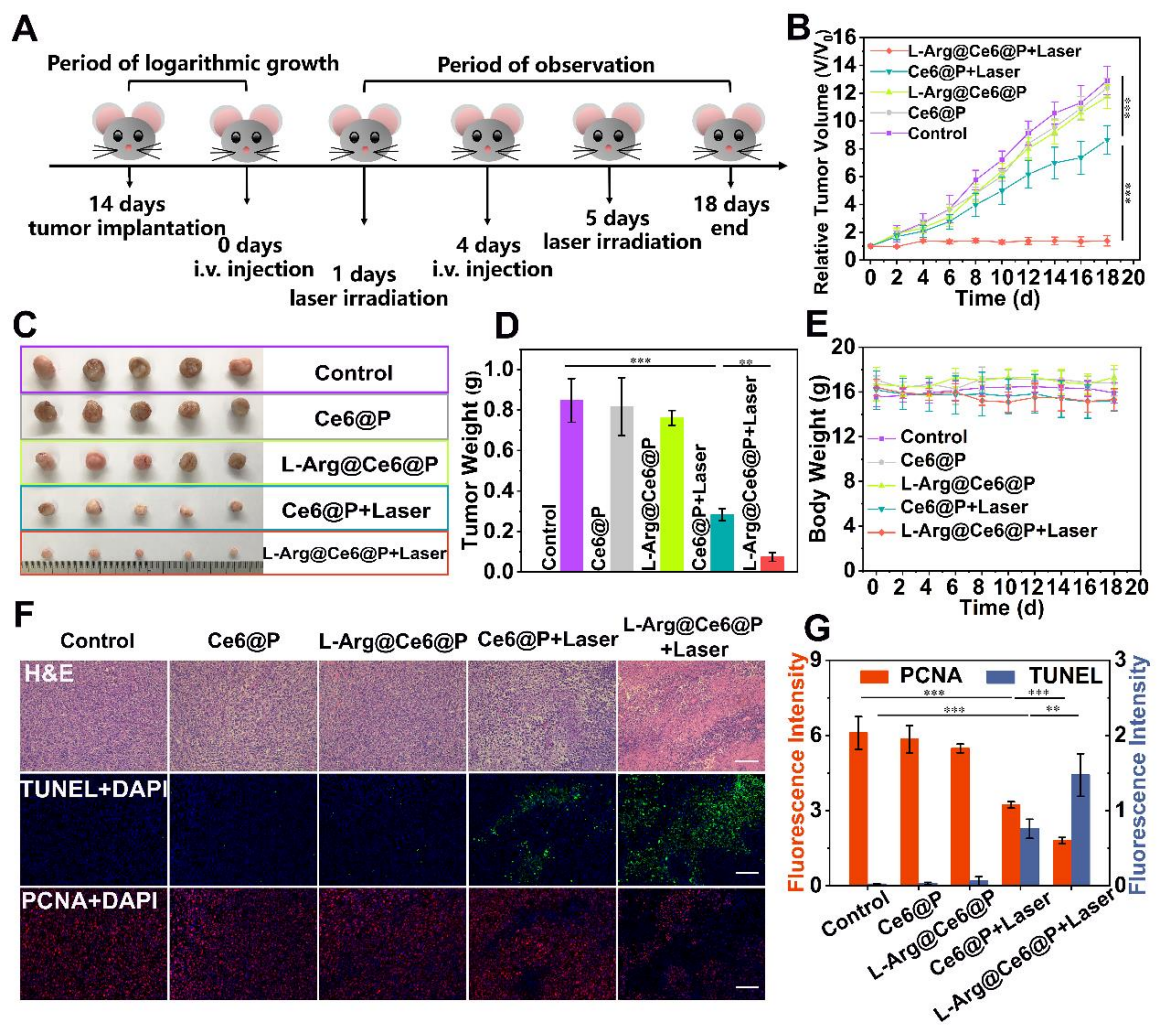
*In vivo* PDT of L-Arg@Ce6@P NPs. (A) Schematic illustration of the L-Arg@ Ce6@P NPs based PDT process. (B) Tumor growth curves of five groups after various treatments. (n = 5, ***p < 0.001). (C) Photographs of tumors dissected from mice of five groups after various treatments. (D)Weight of tumors 18 days post various treatments. (n = 5, **p < 0.01, ***p < 0.001). (E) Body-weight curves of five groups after various treatments. (F) H&E staining, immunochemical staining of TUNEL and PCNA on tumor sections from MDA-MB-231 tumor-bearing mice after various treatments. (scale bar: 100 µm). (G) Mean fluorescence intensity quantitative analysis of PCNA and TUNEL.

The *in vivo* PDT efficacy of different NPs was investigated in tumor-bearing mice, randomly divided into five groups for different treatments. Typically, mice were i.v. injected with NPs at day 0 and day 4, and tumor sites were irradiated (660 nm, 95 mW/cm^2^, 10 min) at 24 h post-injection **(Figure 7A)**. Control, Ce6@P and L-Arg@Ce6@P groups showed no significant tumor growth inhibition effect **(Figure 7B)**. Mice injected with Ce6@P NPs and exposed to laser showed weak anti-tumor efficacy, which is explained by insufficient O_2_ supply which compromises PDT efficacy. In comparison, tumors treated with L-Arg@Ce6@P NPs and laser were clearly reduced, likely because of the reduction in O_2_ consumption and ATP-depletion which sensitize the cells to PDT. Consistently, visualization** (Figure 7C)** and weight **(Figure 7D)** of tumors *in vitro* followed a similar trend, with no significant body weight decrease in the 18-day observation period **(Figure 7E)**.

In addition, we performed HE-staining and immunostaining of TUNEL, PCNA and HE-staining **(Figure 7F)**. HE-staining of tumor cells exposed to L-Arg@Ce6@P and laser showed extensive damage. This was much reduced in the Ce6@P group, and was negligible in the remaining three groups. The trend of cell apoptosis levels observed with TUNEL (green fluorescence) was similar to that of HE-staining, whereas the trend was reversed when using PCNA-staining (red fluorescence) which reflects cell proliferation levels **(Figure 7F)**. Finally, a semi-quantitative analysis of apoptosis and proliferation areas **(Figure 7G)** suggested that the fluorescence intensity of PCNA in the group exposed to Ce6@P with laser was 1.6-fold higher than the L-Arg@Ce6@P with laser group, whereas the fluorescence intensity of TUNEL in the Ce6@P with laser group was half that of the L-Arg@Ce6@P with laser group. These results demonstrate that L-Arg@Ce6@P NPs could enhance PDT efficacy *in vivo*.

### *In vivo* biosafety evaluation

To evaluate the *in vivo* therapeutic safety of L-Arg@Ce6@P NPs, major organs (heart, liver spleen, lung, kidney) obtained from different treatments one day after the second irradiation were stained with H&E** (Figure S14)**. No apparent histopathological abnormalities were observed, suggesting a high therapeutic biosafety of L-Arg@Ce6@P NPs. The potential for short and long terms systemic toxicity of L-Arg@Ce6@P NPs was also evaluated. Other blood biochemical indices showed no significant differences from the control group except for platelet count (PLT), platelet hematocrit (PCT) and aspartate aminotransferase (AST) **(Figure S15)** which may be due to NPs also accumulation in liver and spleen. However, these values were all within the reference range: PLT, 450-1,590 x10^9^/L; PCT, 0.171-0.954%; AST, 55-352 U/L. These results indicate that, despite their accumulation in murine liver and spleen, L-Arg@Ce6@P NPs do not cause adverse effects in those organs, probably because of their rapid metabolism 70, 71. In addition, no significant histopathological changes were observed in different organs **(Figure S16)**, which indicates no toxicity in mice at the tested doses.

## Conclusion

We have constructed a multifunctional nanoplatform that can raise endogenous O_2_ to enhance PDT efficiency and decrease ATP levels that weaken tumor cells. Because of the excellent PA-imaging performance of Ce6, this nanoplatform could be imaged in real time. These results provide new insights to tackle hypoxia through NO inhibition of Mito-AR-related O_2_ metabolism. Compared to traditional PDT, L-Arg@Ce6@ NPs also disturb mitochondrial energy metabolism and renders tumor cells more sensitive to phototherapy by blocking ATP synthesis. Our research demonstrates the potential applicability of this new strategy to amplify the efficacy of PDT.

## Supplementary Material

Supplementary figures and tables.

## Abbreviations

NO: nitric oxide
O_2_: oxygen
PLGA: poly-lactic-co-glycolic acid
Ce6: chlorin e6
H_2_O_2_: hydrogen peroxide
L-Arg: l-Arginine
OXPHOS: oxidative phosphorylation
ATP: adenosine triphosphate
PDT: photodynamic therapy
EPR: enhanced permeability and retention
PA: photoacoustic
NIR: near infrared
DNA: deoxyribonucleic acid
NPs: nanoparticles
ROS: reactive oxygen species
Mito-AR: mitochondrial aerobic respiration
ETC: electron transport chain
iNOS: inducible nitric oxide synthases
C*c*O: cytochrome *c* oxidase
PVA: polyvinyl alcohol
CHCl_3_: chloroform
SOSG: singlet oxygen sensor green
TEM: transmission electron microscope
SEM: scanning electron microscope
MMP: mitochondrial membrane potential
HbO_2_: oxygenated hemoglobin
HE: hematoxylin & eosin
PCNA: proliferating cell nuclear antigen
TUNEL: terminal deoxynucleotidyl transferase-mediated dUTP-biotin nick end labeling
