# Supplementary material for: Increased photodynamic therapy sensitization in tumors using a nitric oxide-based nanoplatform with ATP-production blocking capability
Source: Theranostics. 2021 Jan 1;11(4):1953–69. doi: 10.7150/thno.52997 (PMC7778583; doi:10.7150/thno.52997)
Supplement: Supplementary file 1 — Supplementary figures and tables. [file thnov11p1953s1.pdf]

Supporting Information

Increased photodynamic therapy sensitization in tumors using a nitric oxide-based nanoplatform with ATP-production blocking capability

Qinyanqiu Xiang[1], Bin Qiao[1], Yuanli Luo[1], Jin Cao[1], Kui Fan[2], Xinghua Hu[3], Lan Hao[1], Yang Cao[1], Qunxia Zhang[1]* and Zhigang Wang[1]*

1. Institute of Ultrasound Imaging, Second Affiliated Hospital of Chongqing Medical University, Chongqing, 400010, P. R. China.

2. Department of Nephrology, Second Affiliated Hospital of Chongqing Medical University, Chongqing, 400010, P. R. China.

3. Department of Neurosurgery, Second Affiliated Hospital of Chongqing Medical University, Chongqing, 400010, P. R. China.

*Corresponding Author: 303507@hospital.cqmu.edu.cn (Zhigang Wang)

300771@hospital.cqmu.edu.cn (Qunxia Zhang)

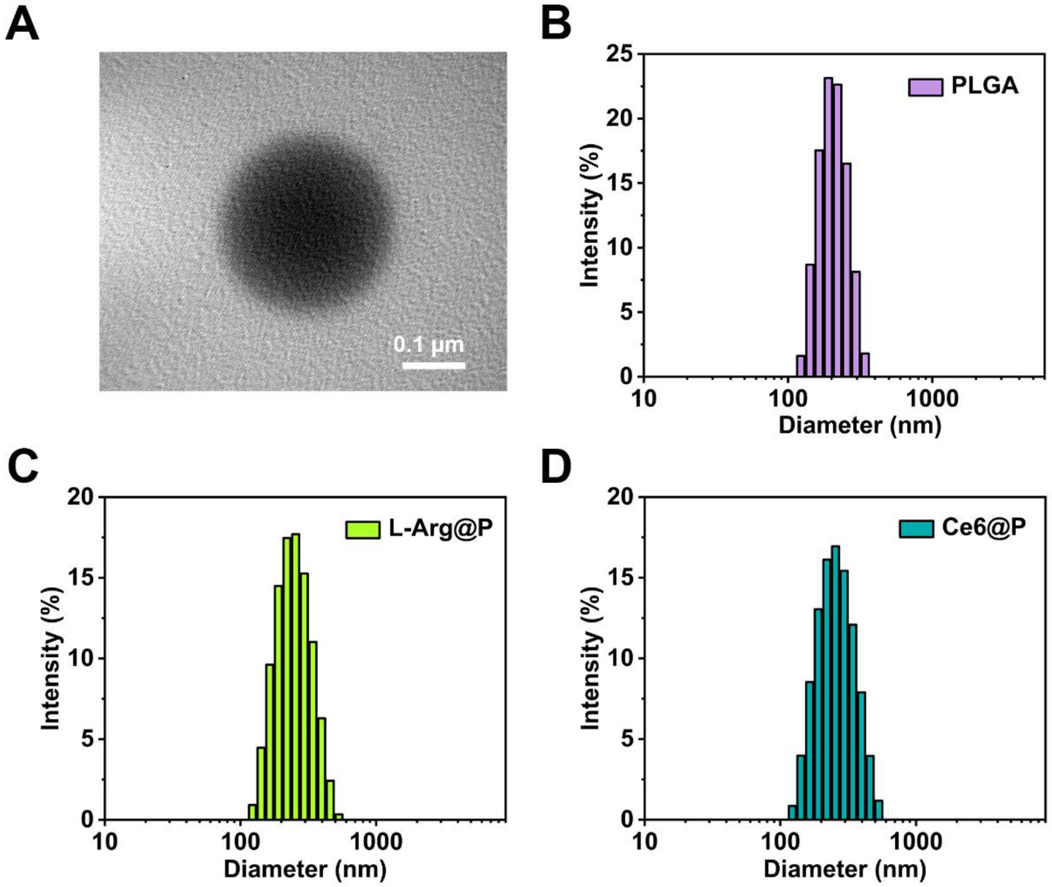

Figure S1. (A) The TEM of L-Arg@Ce6@P NPs (scale bar: 0.1 μm). (B)-(D) Size distribution of different NPs as measured by DLS. (B) PLGA, (C) L-Arg@P, (D) Ce6@P.

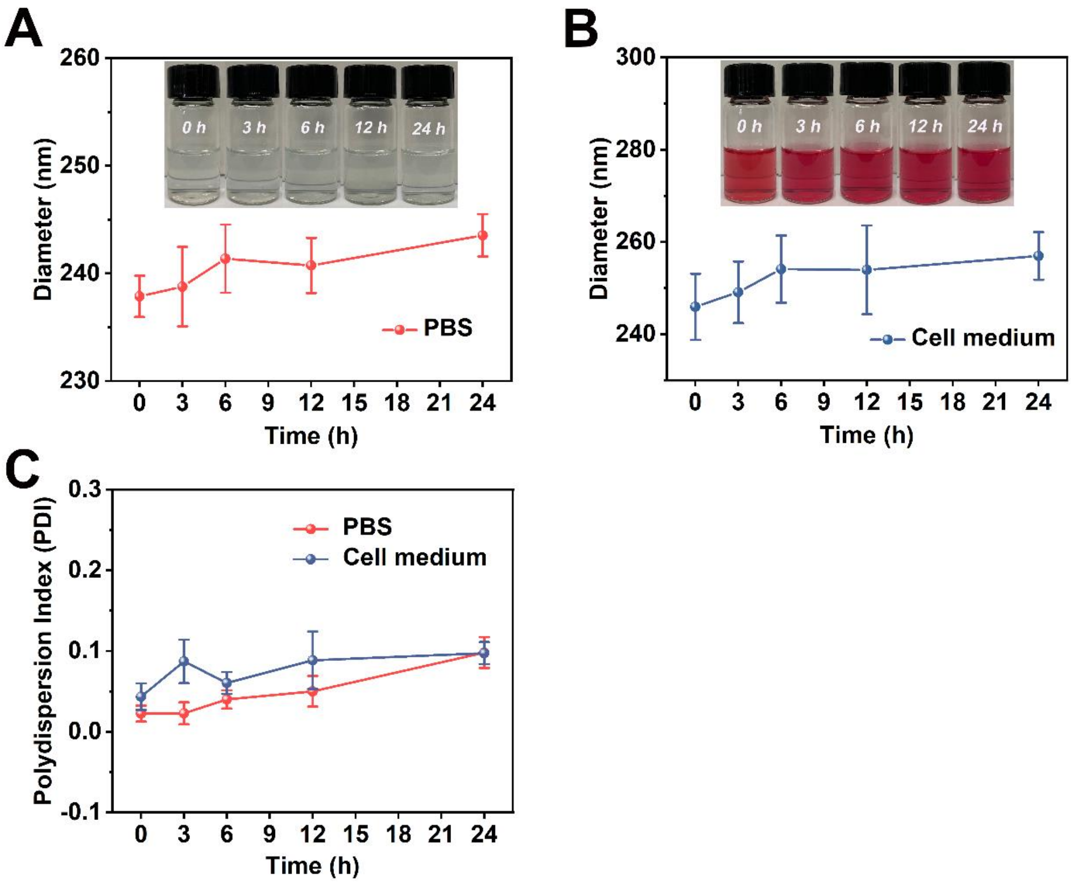

Figure S2. Hydrodynamic diameters of L-Arg@Ce6@P NPs (0.5 mg/mL) with prolonged time duration in PBS (A) and DMEM cell culture medium containing 10% serum (B). (C) Corresponding PDI of size distribution.

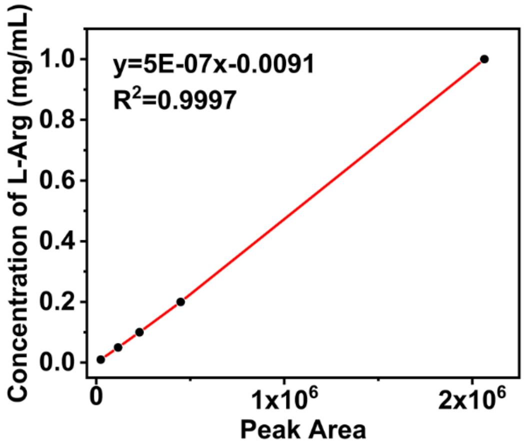

Figure S3. The standard curve of free L-Arg measured by HPLC.

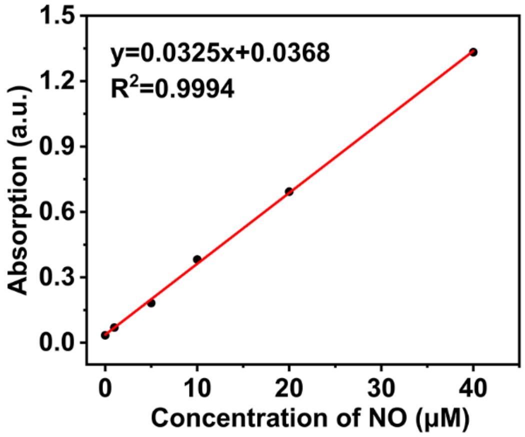

Figure S4. The standard curve of NO measured by the multimode reader at 540 nm with the Griess Reagent.

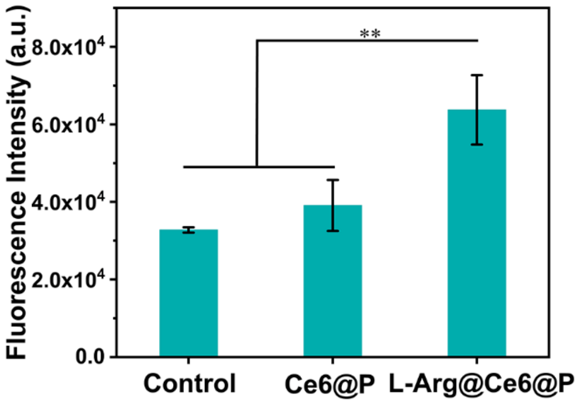

Figure S5. Mean fluorescence intensity of DAF-FM DA in different groups quantified by flow-cytometry. (n = 3, **p < 0.01).

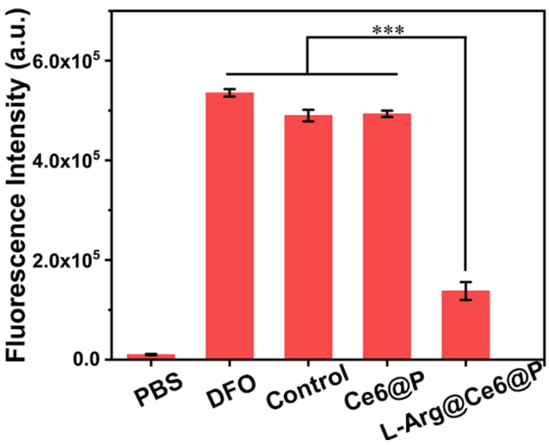

Figure S6. Mean fluorescence intensity of ROS-ID in different groups analyzed by flow-cytometry. (n = 3, ***p < 0.001).

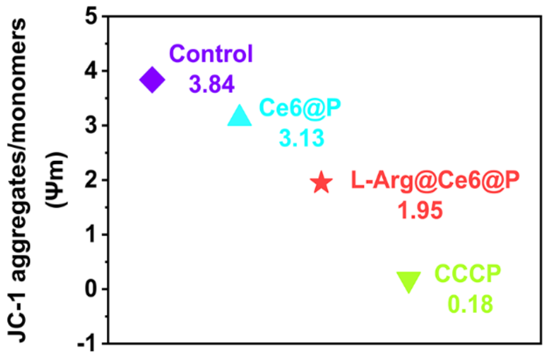

Figure S7. Mitochondrial depolarization (Ψm) of different groups.

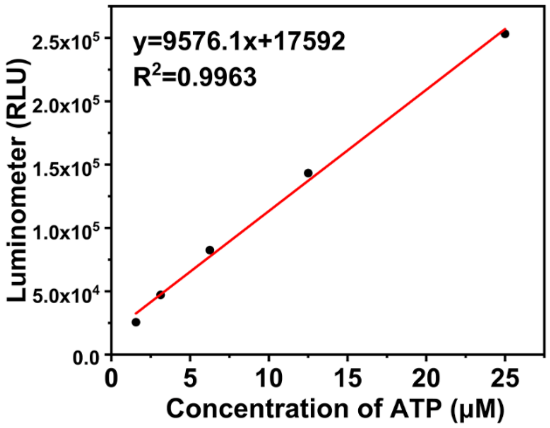

Figure S8. The standard curve of ATP measured by luminometer in multimode reader with the ATP assay kit.

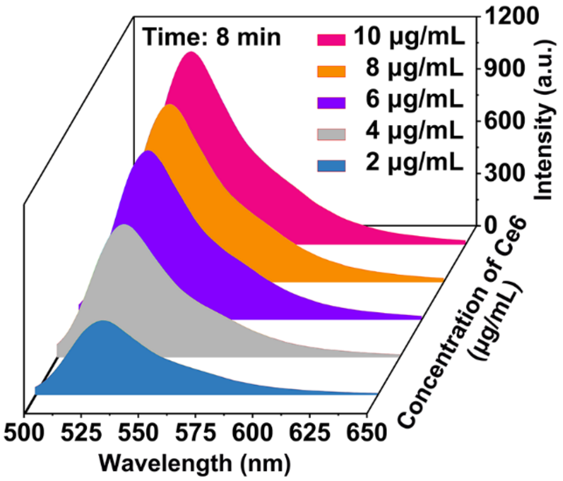

Figure S9. Concentration-dependent $^1O_2$ generation of L-Arg@Ce6@P NPs irradiated by 660 nm laser (5 mW cm^{-2}) for 8 min.

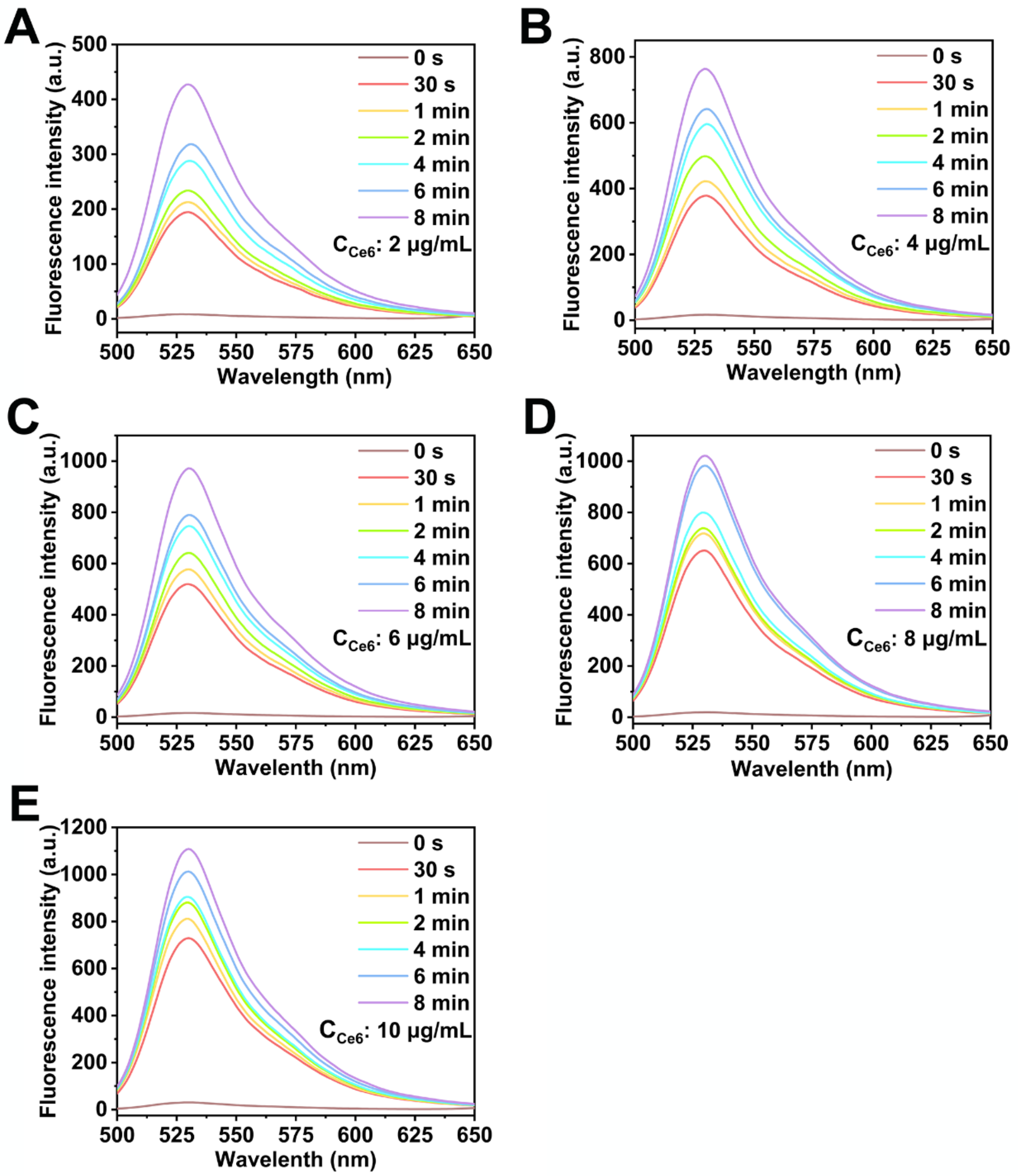

Figure S10. (A)-(E) Time-dependent $^1O_2$ generation of L-Arg@Ce6@P NPs at different concentrations irradiated by 660 nm laser (5 mW cm^{-2}).

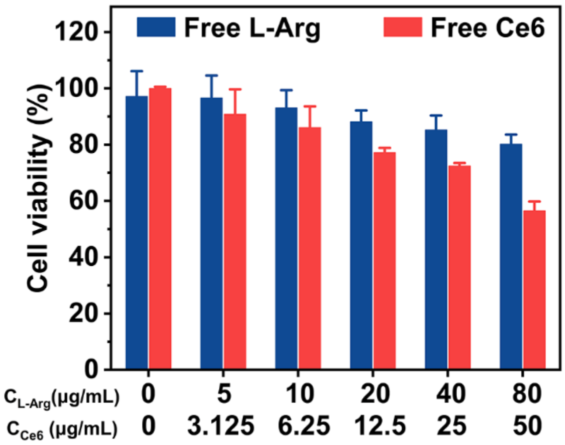

Figure S11. 24 h cytotoxicity of free L-Arg and free Ce6.

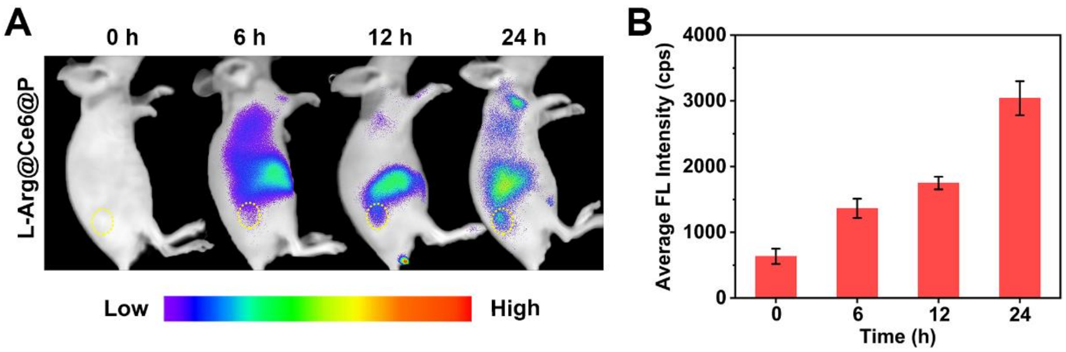

Figure S12. (A) Biodistribution of L-Arg@Ce6@P NPs *in vivo* at 0, 6, 12 and 24 h post-injection. (B) Fluorescence quantitative analysis of tumor tissue.

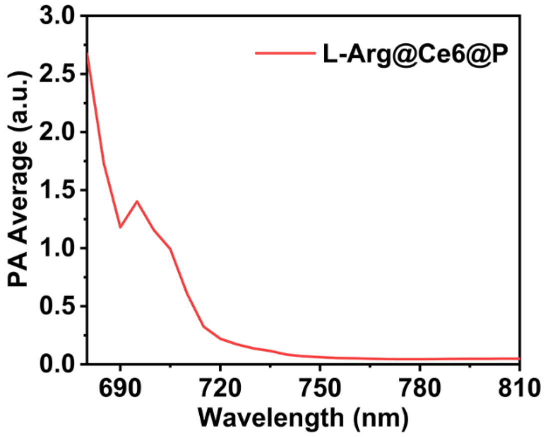

Figure S13. PA-signal changes of L-Arg@Ce6@P NPs as irradiated by a laser at the wavelength range of 680-810 nm.

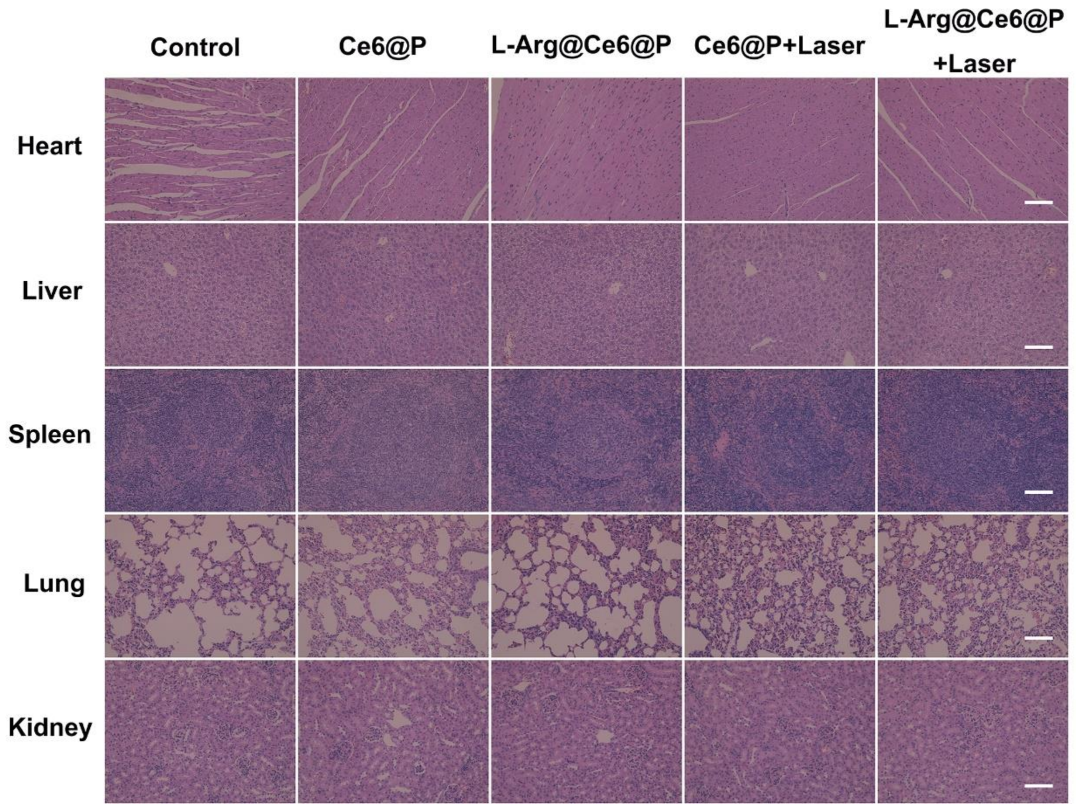

Figure S14. H&E staining of the major organs (heart, liver, spleen, lung, and kidney) of tumor-bearing mice after different treatments. (scale bar: 100 μm).

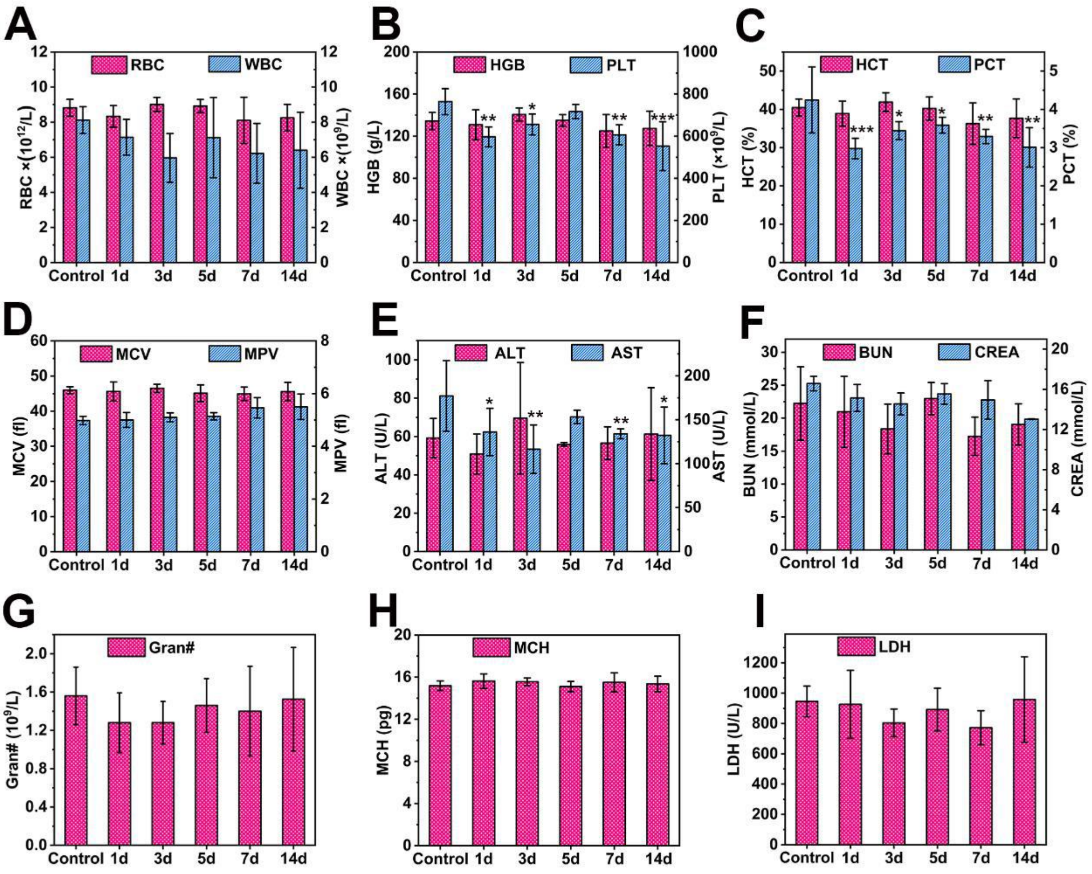

Figure S15. (A)-(I) Blood biochemical indexes analysis of BALB/c mice from the control group and the experimental groups 1, 3, 5, 7 and 14 days post intravenous injection of L-Arg@Ce6@P NPs. (n = 5, * $P< 0.05$, ** $P< 0.01$, ***$P< 0.001$, vs. Control group).

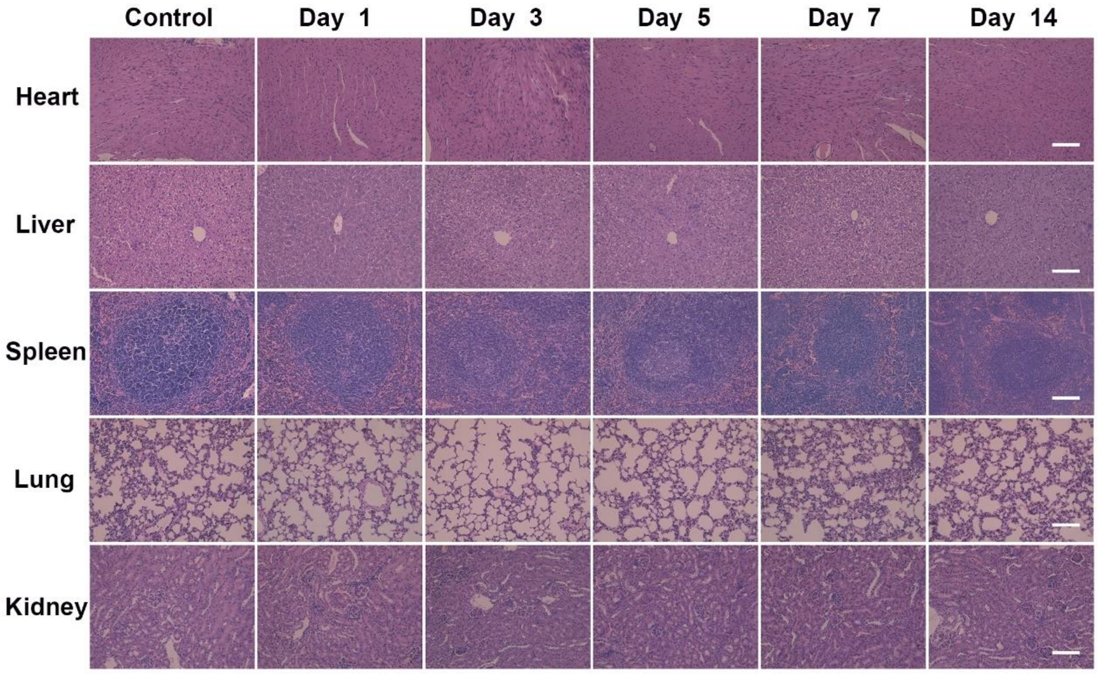

Figure S16. H&E staining of major organs from the control group and the experimental groups 1, 3, 5, 7 and 14 days post intravenous injection of L-Arg@Ce6@P NPs. (scale bar: 100 μm).
